# Live Fast, Die Old: Lowering Insulin Signalling During Development Can Speed Growth, Increase Reproduction and Lifespan, but Impair Environmental Sensing and Harm Offspring in *Caenorhabditis elegans*

**DOI:** 10.1111/acel.70783

**Published:** 2026-10-23

**Authors:** Sara D. Irish, Annabel Kimberley, Peter Lenart, Hanne Carlsson, Anna Braybrooke, Benjamin Towbin, Simone Immler, Alexei A. Maklakov

**Affiliations:** ^1^ School of Biological Sciences University of East Anglia, Norwich Research Park Norwich UK; ^2^ Institute of Cell Biology University of Bern Bern Switzerland

**Keywords:** ageing, ecological physiology, life‐history evolution, senescence

## Abstract

Genetic trade‐offs can constrain life‐history evolution resulting in the evolution of ageing. Reduced insulin/insulin‐like signalling (rIIS) improves somatic maintenance and dramatically increases lifespan in *Caenorhabditis elegans* , but the effects on fitness are unclear. Here we used a novel experimental approach to show that, contrary to the prevailing paradigm, rIIS via *daf‐2* RNAi during development can improve development, reproduction, survival, individual fitness, and population growth rate in the laboratory and semi‐natural conditions. However, developmental rIIS extends lifespan and improves fitness only in some temperatures, which explains why our results differ from previous findings. Furthermore, while developmental rIIS can improve a suite of life‐history traits in parents, it slows down offspring development. Finally, because rIIS interferes with environmental sensing and compromises adaptive developmental plasticity in response to stress, a central part of *C. elegans* life history in nature, the ability to sense the environment correctly during development comes at the cost of accelerated senescence. These results suggest the complexity of gene‐by‐environment interactions during development imposes constraints on molecular signalling networks leading to genetic trade‐offs across the life course that natural selection in late‐life is unable to resolve. However, these constraints can be resolved by artificially fine‐tuning gene expression at different ages.

## Graphical abstract

Developmental reduction of insulin/IGF‐1 signalling via *daf‐2* RNAi accelerates development, increases early reproduction, fitness, and lifespan in *C. elegans,* but compromises environmental sensing. Reduced IIS promotes inappropriate offspring dauer entry and delays recovery from developmental arrest. Selection optimising complex molecular signalling networks for early‐life performance can be a cause of ageing.

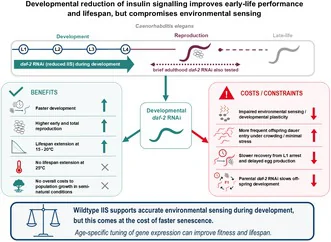

## 1 Introduction

Ageing is a complex and multifaceted process, and the evolutionary mechanisms behind its nearly universal presence remain incompletely resolved (Slade et al. 2024; Cohen et al. 2020; Gems and Kern 2024). The decline in the force of natural selection with age (so‐called ‘selection shadow’) (Medawar 1952; Williams 1957; Hamilton 1966) leads to accumulation of late‐acting deleterious alleles (‘mutation accumulation’) (Medawar 1952) and selection for alleles whose effects on fitness are beneficial in early‐life but detrimental in late‐life (‘antagonistic pleiotropy’) (Williams 1957). However, these population genetic models do not offer insight into the physiological processes that result in trait senescence, and ultimately shape organismal life histories. The developmental theory of ageing (DTA) argues that ageing results from suboptimal regulation of gene expression in adulthood, making senescence a by‐product of developmental processes that are strongly selected and tightly regulated (de Magalhães and Church 2005; Maklakov and Chapman 2019; Lemaître et al. 2024). In broader evolutionary terms, the DTA maintains that ageing results from the age‐related decline in the strength of selection, which leaves physiological processes finely tuned for early‐life development, growth and reproduction but insufficiently tuned for late‐life function (Lemaître et al. 2024).

The DTA follows from a simple observation that the physiological needs of an organism change with age leading to selection on age‐specific gene expression (de Magalhães and Church 2005; Maklakov and Chapman 2019). While age‐specific gene expression is well documented, the DTA maintains that ‘selection shadow’ will result in less optimised gene expression in adulthood, because the force of selection is maximal during development and declines gradually after the onset of reproduction (Lemaître et al. 2024). The decline in the force of natural selection is predicted to lead to either higher gene expression than optimal for fitness (‘hyperfunction’) or lower gene expression than optimal for fitness (‘hypofunction’) with advancing age (Maklakov and Chapman 2019; Lemaître et al. 2024). The DTA makes the specific prediction that it is possible to modify gene expression in adulthood, but not in development, to slow down senescence and increase fitness (Maklakov and Chapman 2019; Lemaître et al. 2024).

Here we test the DTA by focusing on environment‐sensing insulin/insulin‐like signalling (IIS) pathway. The IIS pathway is highly evolutionarily conserved and plays a key role in shaping organismal life history in response to diverse environmental cues (Kenyon 2001, 2005; Fontana et al. 2010). In *Caenorhabditis elegans* nematodes, downregulation of the IIS pathway via knockdown of the *daf‐2* receptor substantially extends lifespan, more than two times as long as wildtype lifespan, in knockout mutants (Kenyon et al. 1993) or through adulthood knockdown with RNAi (Dillin et al. 2002; Lind et al. 2019). However, knockout *daf‐2* mutants suffer fitness costs in laboratory conditions, including compromised development, delayed reproduction, and lower offspring number (Gems et al. 1998). Similarly, *daf‐2* RNA interference (RNAi) knockdown during development appears to be detrimental for early reproduction resulting in reduced fitness (Dillin et al. 2002; Jenkins et al. 2004). These results suggest that strong selection in early life has optimised wildtype IIS regulation during development, and early‐life changes in IIS levels carry fitness costs, whereas when adulthood regulation is changed no such costs are found (Dillin et al. 2002; Lind et al. 2019).

Importantly, the IIS pathway plays a critical role in the regulation of developmental plasticity in response to environmental cues in *C. elegans* (Dillin et al. 2002; Kimura et al. 1997). Normal development of *C. elegans* requires undergoing four larval stages before reaching sexual maturity (Byerly et al. 1976). Before their second larval stage, *C. elegans* have the option to carry on to sexual maturation or enter a non‐feeding, non‐reproductive and highly stress resistant diapause state known as dauer (Cassada and Russell 1975). They can enter dauer state in response to stressful conditions, especially low resource availability and overcrowding, and remain this way for several months (Golden and Riddle 1982). This developmental plasticity is ecologically important for *C. elegans* , as in nature, they survive on ephemeral food sources that run out rapidly as populations grow (Schulenburg and Félix 2017). Entering dauer state allows migration to new food sources and continuation on to sexual maturity in a new, resource‐laden environment (Golden and Riddle 1984). Because of the key role of IIS in regulating developmental plasticity in response to the environment, downregulation of IIS may result in inappropriate responses to environmental conditions experienced by larval nematodes (Cassada and Russell 1975; Gems et al. 1998). For example, environmental cues, such as high temperature and low food availability, experienced in the first ten to sixteen hours post‐hatching can decrease the production of agonistic insulin‐like peptides (ILPS) which downregulate IIS via the DAF‐2 receptor to promote dauer formation (Li et al. 2003; Hung et al. 2014; Cornils et al. 2011). Downregulation of *daf‐2* would cause similar effects to those of attenuated ILPS, and potentially amplify them, thus facilitating dauer formation in the environments that allow normal reproduction (Fielenbach and Antebi 2008). Given that it takes approximately 1 day to enter dauer, and another day minimum to recover from dauer and successfully develop into a sexually mature adult (Cassada and Russell 1975; Golden and Riddle 1984; Hu 2018; Karp 2018), this could incur massive fitness costs by delays in or even prevention of reproduction. In fact, increased rates of dauer entry in response to elevated temperature were observed in *C. elegans* following *daf‐2* RNAi (Dillin et al. 2002) or DAF‐2 degradation using auxin‐induced degron (AID) approach (Venz et al. 2021). Recent hypothesis proposed that, given the primary role of IIS in environmental sensing, and in integration of environmental cues into adaptive phenotypic responses, downregulation of IIS will compromise adaptive phenotypic plasticity (Regan et al. 2020). Here we integrate this proximate hypothesis with predictions derived from the DTA. Specifically, we predict that downregulation of IIS during development will increase fitness in stable environments but will compromise the ability of *C. elegans* to sense the environment correctly, resulting in impaired developmental plasticity.

We combine novel laboratory and outdoor experimental approaches to test this prediction (Figure 1A,B). By performing fitness and lifespan assays following IIS knockdown during development, we tested whether IIS regulation is optimised for early life, as predicted by the DTA due to strong early‐life selection pressures. As our daf‐2 RNAi treatment did not facilitate increased dauer or arrest stage entry in our standard laboratory conditions, using different environmental conditions allowed us to test different levels of stress on arrest entry and recovery rates. Furthermore, by performing our assays in varying environmental conditions, both in the laboratory and outdoors, we were able to assess whether there are detrimental fitness effects of IIS downregulation in more ecologically realistic environments. If such fitness costs were found when IIS knockdown occurs in adulthood, this would indicate that the beneficial effects previously discovered may be a laboratory artefact, as has been previously suggested (Briga and Verhulst 2015). However, if no such costs were found, this would indicate that adulthood IIS levels can be optimised, even in challenging conditions, further supporting the predictions of the DTA. For developmental IIS knockdown, we would expect environmental conditions to be the determining factor for changes in fitness.

**FIGURE 1:**
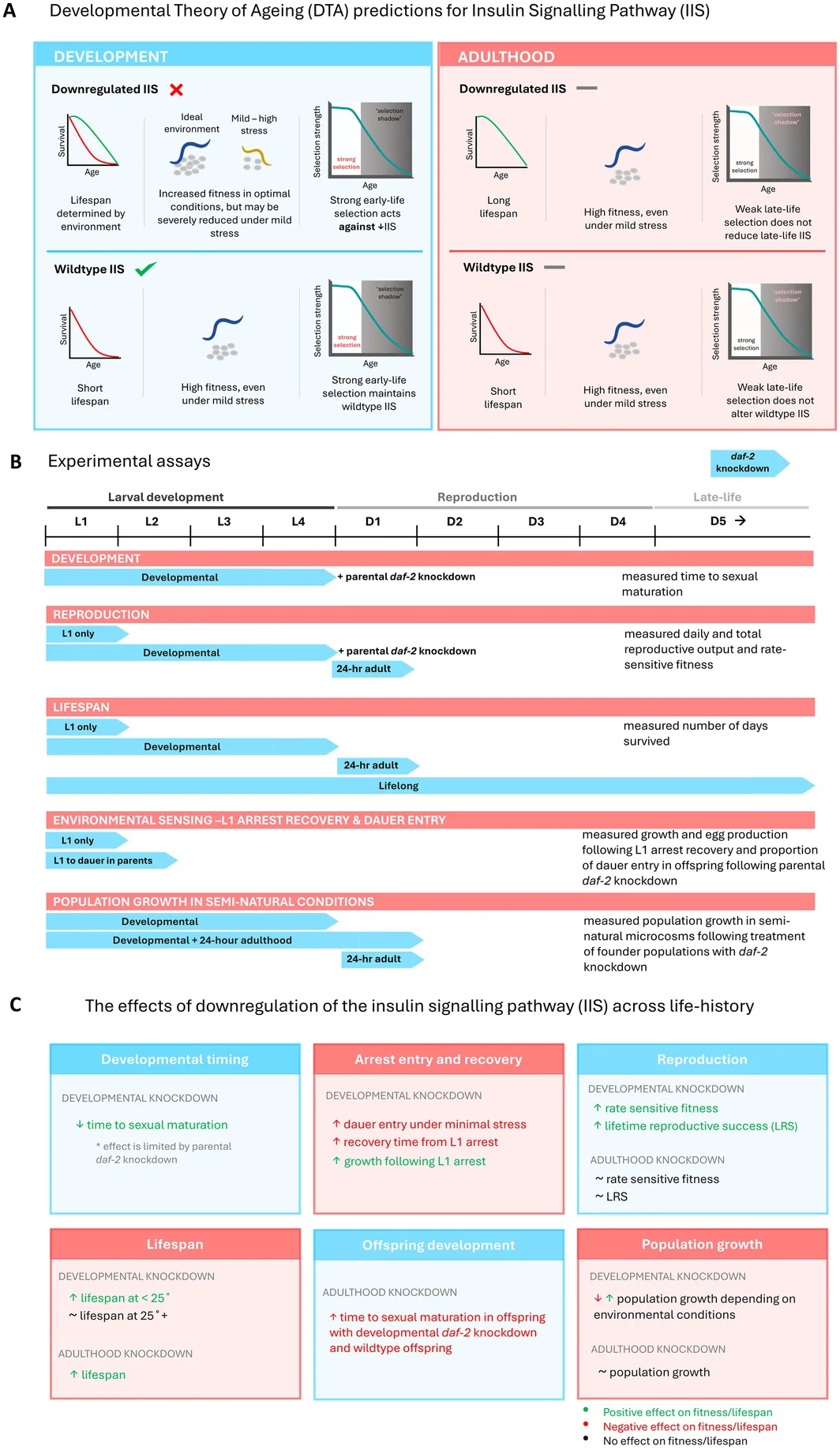
Testing the Developmental Theory of Ageing (DTA) through age‐specific insulin signalling (IIS) downregulation in the laboratory and in semi‐natural conditions. (A) describes the predictions of the DTA for age‐specific regulation of the IIS pathway. (B) shows experimental assays, with a description of the *daf‐2* RNAi treatment timing to downregulate IIS, with an explanation of what was measured in each assay. (C) provides an overview of the results for each assay type and how these relate to fitness, with positive fitness benefits shown in green and fitness costs in red. In (C), arrows indicate the direction of the treatment effect on the trait described, with ~ indicating no detected effect.

First, we show that developmental downregulation of IIS accelerates development which can result in increases for early‐life reproduction to improve individual fitness and population growth rate, but the magnitude of these effects is temperature‐dependent. Second, we show that reduced IIS in parents slows offspring development and increases the propensity of offspring to enter reproductive diapause under the conditions that allow growth and reproduction (Figure 1C). We discuss the implications of these findings for the evolutionary theory of ageing and suggest that strong selection during development on regulatory signalling pathways with broad pleiotropic effects constrains late‐life performance.

## 2 Results

### 2.1 Brief, Transient IIS Downregulation During Development or Adulthood Increases Lifespan

Constant post‐developmental *daf‐2* RNAi approximately doubles lifespan in *C. elegans* (Treatment: $\chi_{3}^{2}$ = 182.658, *p* < 0.0001, Figure 2A, Table S1). We also found that even a brief, transient treatment of *daf‐2* RNAi during adulthood increases lifespan by up to 40% (*p* < 0.0001, Figure 2A, Table S1). Transient *daf‐2* knockdown also increased lifespan when treatment occurred during development for just 24 h (Treatment: $\chi_{3}^{2}$ = 182.658, *p* < 0.0001, Figure 2A, Table S1) or for the entirety of development ($\chi_{1}^{2}$ = 6.382, *p* = 0.011, Figure 2C, Table S2), but the magnitude of the effect was temperature‐dependent. Median lifespan was increased by almost 35% following developmental *daf‐2* knockdown at 15°C ($\chi_{1}^{2}$ = 49.083, *p* < 0.0001, Figure 2B, Table S3), but developmental *daf‐2*‐mediated lifespan extension was lost at 25°C ($\chi_{1}^{2}$ = 0.025, *p* = 0.848, Figure 2D, Table S4). The lifespan extension phenotype persisted long after *daf‐2* expression levels return to that of controls, within 48 h after removal from RNAi (Figure 2E, Table S5).

**FIGURE 2:**
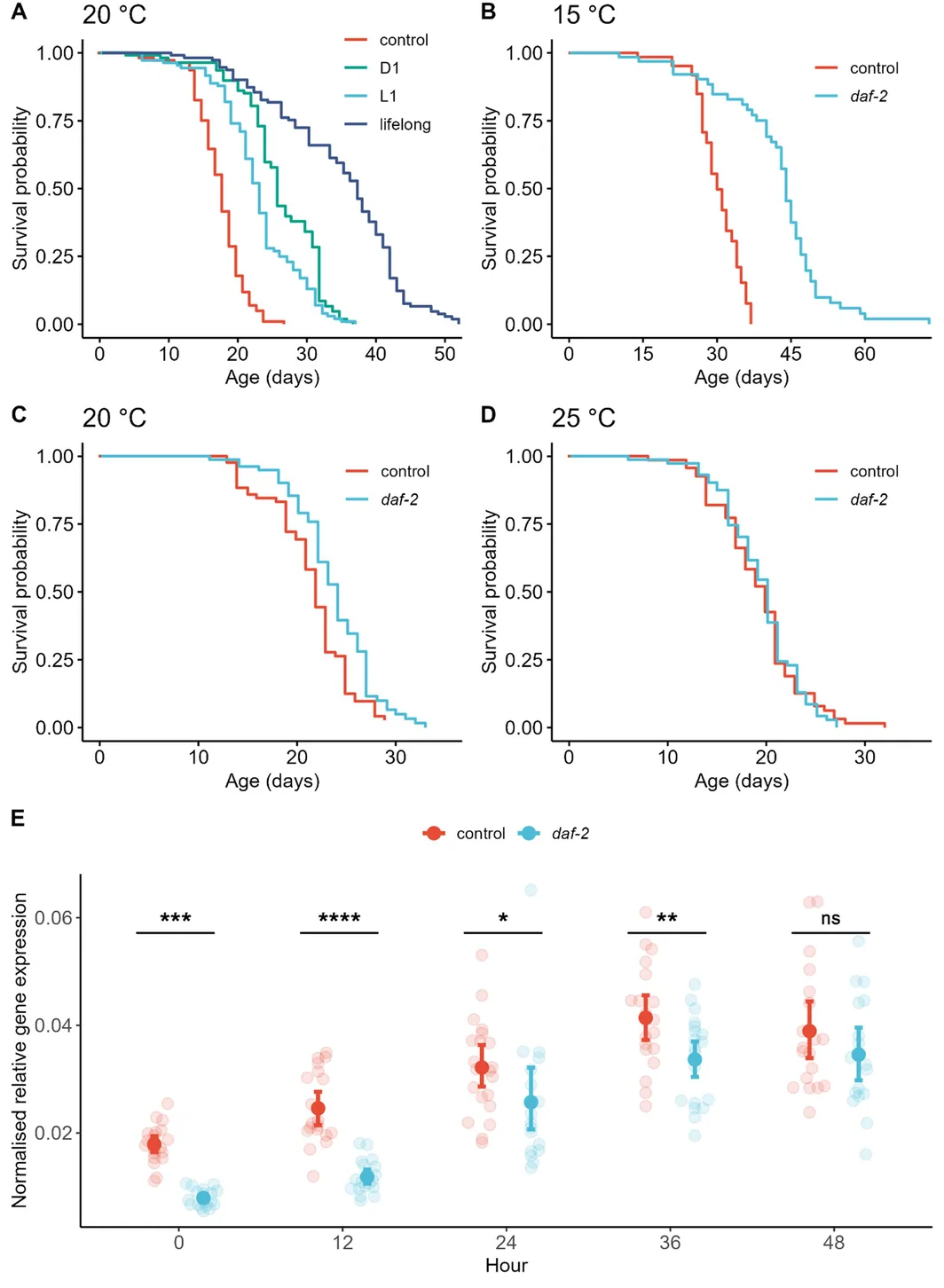
Brief, transient IIS downregulation extends lifespan, even after *daf‐2* gene expression levels return to that of controls: (A) Survival probability of nematodes following transient, 24‐h downregulation of insulin signalling (IIS) via the *daf‐2* receptor starting from hatching (*n* = 110) or upon reaching sexual maturity (*n* = 114) compared to lifelong downregulation (*n* = 115) and controls (*n* = 115). Survival probability following IIS downregulation during the entire developmental period (from hatching until sexual maturity) at (B) 15°C (*n*_control_ = 73, *n*_*daf‐2*_ = 65), (C) 20°C (*n*_control_ = 92, *n*_*daf‐2*_ = 83), and (D) 25°C (*n*_control_ = 73, *n*_*daf‐2*_ = 80). (E) Relative gene expression of *daf‐2* normalised to housekeeping gene *Actin‐1* 0–48 h post 24‐h *daf‐2* downregulation on day 1 of adulthood and empty vector (ev) controls (*n* > 15 per treatment per time point).

### 2.2 Developmental IIS Downregulation Can Speed Up Development, but Parental Knockdown Ameliorates This Effect

Developmental *daf‐2* knockdown from hatching to sexual maturity resulted in faster development at 15°C, 20°C, and 25°C compared to controls (15°C: $\chi_{1}^{2}$ = 30.914 *p* < 0.0001; 20°C: $\chi_{1}^{2}$ = 111.000, *p* < 0.0001; 25°C: $\chi_{1}^{2}$ = 6.055, *p* = 0.014; Figure 3A–C, Table S6). Performing this experiment across two laboratories with unique experimental setups (Experiments 1 and 2) and including parental *daf‐2* knockdown during the reproductive period yielded slightly different results. When parents of developmental *daf‐2* offspring also experienced *daf‐2* knockdown during reproduction, parental treatment slowed development ($\chi_{1}^{2}$ = 10.950, *p* = 0.001), but developmental treatment still reduced development time ($\chi_{1}^{2}$ = 22.507, *p* < 0.0001) in Experiment 1 (Figure 3D, Table S7). In Experiment 2, the effect of parental *daf‐2* knockdown was dependent on offspring treatment (Parent*Offspring: $\chi_{1}^{2}$ = 4.915, *p* = 0.027), with the slowing of development time due to parental *daf‐2* knockdown appearing stronger in control offspring (Figure 3E, Table S7). There was no apparent effect of offspring treatment on development time within parental treatments (Table S7).

**FIGURE 3:**
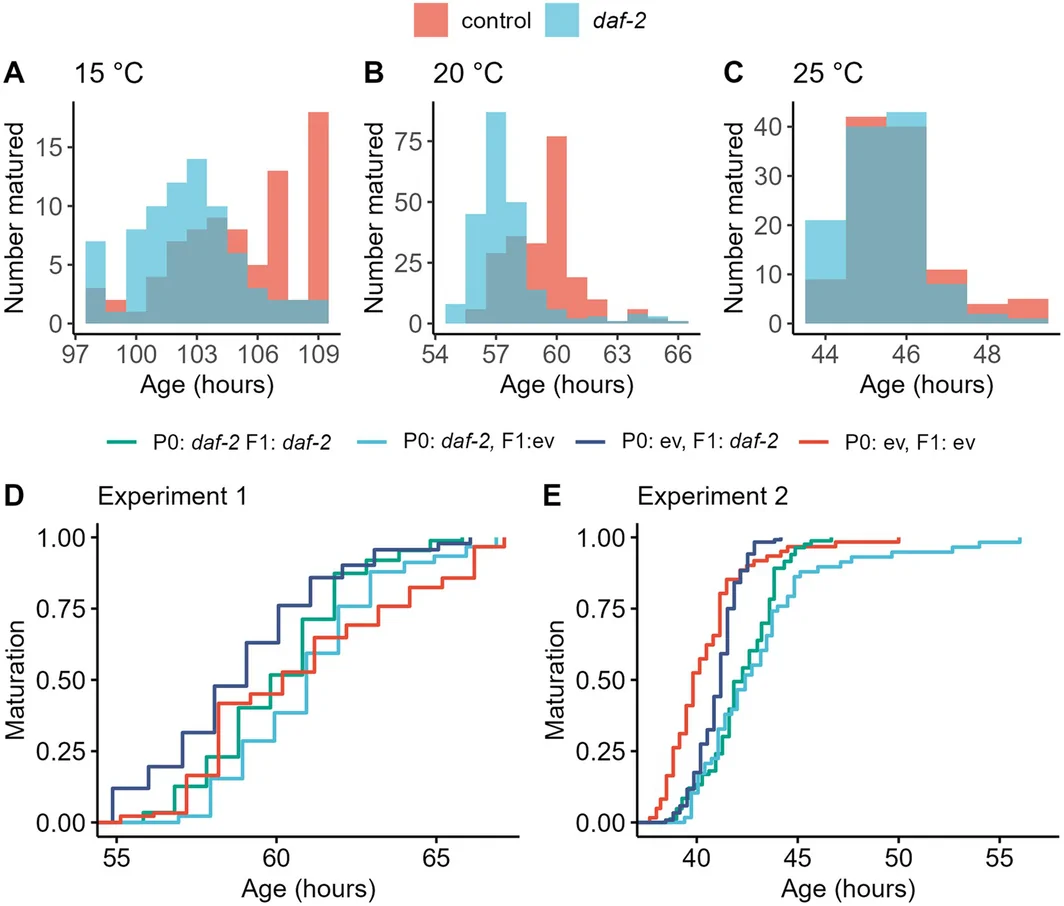
Developmental IIS downregulation effects on development speed are mediated by both environmental temperature and parental IIS regulation: Numbers of individuals matured per hour following downregulation of insulin signalling via *daf‐2* knockdown during development or empty vector (ev) controls at (A) 15°C (*n*_ev_ = 80, *n*_daf_ = 77), (B) 20°C (*n*_ev_ = 220, *n*_daf_ = 224), and (C) 25°C (*n*_ev_ = 111, *n*_daf_ = 115). Proportion of individuals matured following both parental and offspring treatments of *daf‐2* or control bacteria in 20°C in two experiments from different laboratories in (D) Experiment 1 (*n* > 85 for all treatments) and in (E) Experiment 2 (*n* > 58 for all treatments). In Experiment 1, maturation was identified through the visual identification of vulva eversion. For Experiment 2, maturation was determined as the final larval moult (M4) using growth curves.

### 2.3 Developmental IIS Downregulation Improves Reproduction

Individuals that experienced *daf‐2* RNAi during development experienced improved early reproduction (Treatment*Day^2^: $\chi_{1}^{2}$ = 5.456, *p* = 0.02, Figure 4A), higher lifetime reproductive success (LRS; *F*_1,215_ = 10.003, *p* = 0.002), and higher individual fitness (*F*_1,220_ = 9.891, *p* = 0.002, Figure 4B, Table S8) than controls when all individuals from a randomly pre‐selected cohort are included in reproductive assays. When analysing this data only including individuals who matured on or after the peak of maturation of controls (individuals likely to be included in an experimental setup where nematodes are selected based on their larval stage at a given setup time), the improvements of developmental *daf‐2* RNAi to early reproduction (Treatment: $\chi_{1}^{2}$ = 0.014, *p* = 0.905, Table S9, Figure 4C) and LRS (*F*_1,87_ = 0.039, *p* = 0.845, Table S9) were lost. Additionally, a trend toward lower individual fitness (*F*_1,89_ = 0.664, *p* = 0.066, Table S9, Figure 4D) was observed for developmental *daf‐2* knockdown individuals. However, the beneficial effects of developmental *daf‐2* knockdown on LRS and individual fitness were lost under lower (15°C) and higher (25°C) temperatures, but costs were not observed either (15°C: Figure 4E,F, Table S10; 25°C: Figure 4G,H, Table S11). Including all individuals in a cohort did not affect the reproduction of nematodes following *daf‐2* RNAi during 24‐h in adulthood (Figure 4I,J, Table S12). For offspring with developmental *daf‐2* knockdown in 20°C, parental *daf‐2* treatment during reproduction had an interactive effect with offspring treatment on age‐specific reproduction ($\chi_{2}^{2}$ = 16.527, *p* = 0.0003, Figure 4K), but only offspring *daf‐2* knockdown had a positive effect on LRS (*F*_1,173_ = 8.046, *p* = 0.005) and individual fitness (*F*_1,173_ = 14.674, *p* = 0.0002, Figure 4L, Table S13). Overall, we found that reduced IIS during adulthood carries no costs to reproduction, and that reduced IIS during development improves both early reproduction and total reproductive output. However, parental IIS knockdown reduces the beneficial effects of developmentally reduced IIS.

**FIGURE 4:**
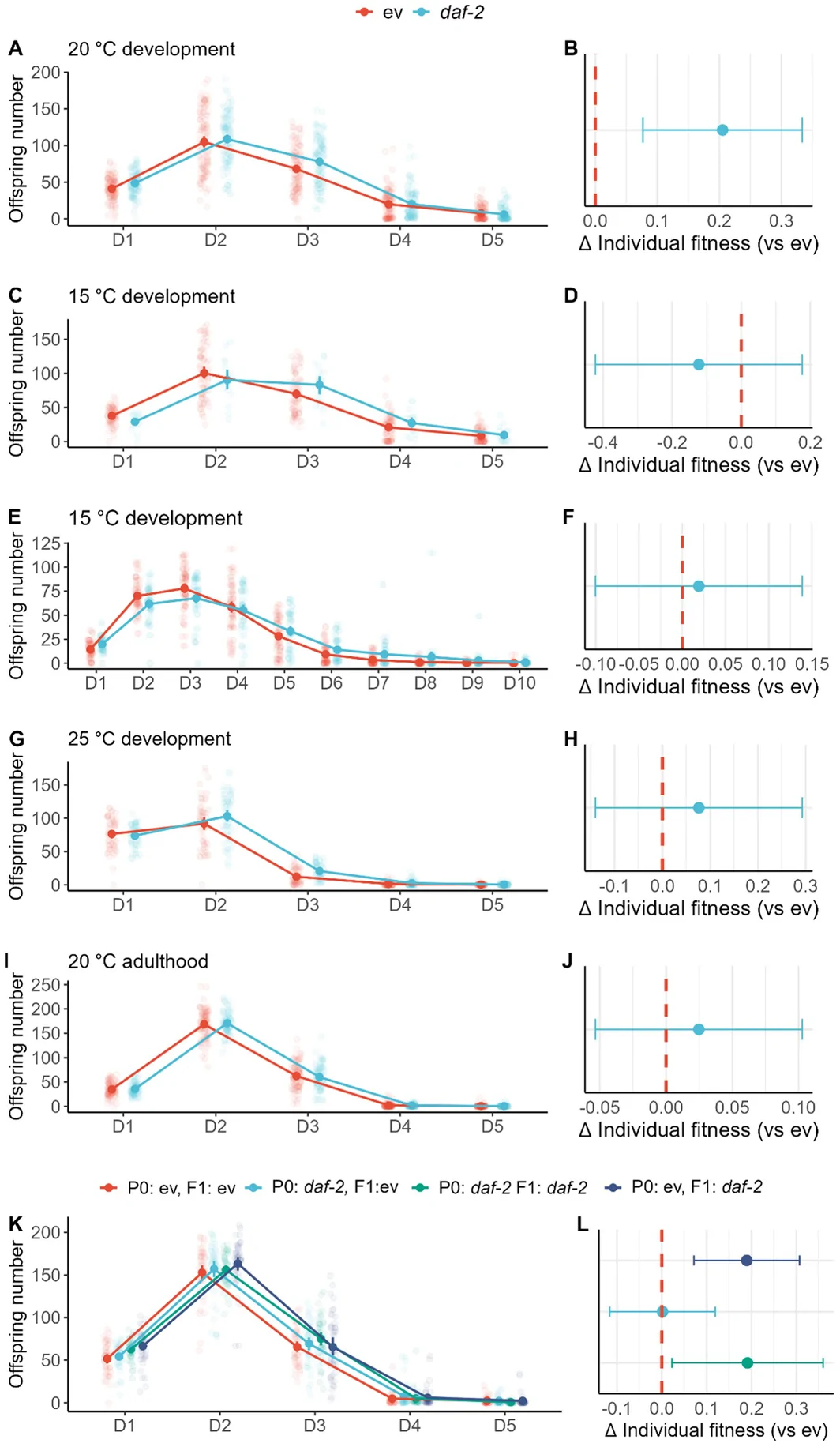
IIS downregulation during development improves early reproduction and lifetime reproductive success in a temperature‐dependent manner: Age‐specific reproduction, shown as the mean number of offspring produced per day, and individual fitness, shown as the mean difference from a linear model, following *daf‐2* downregulation during development when housed at (A, B) 20°C (*n*_ev_ = 107, *n*_daf_ = 110), and (C, D) when only including individuals that matured on or after the peak of maturation in controls from A and B (*n*_ev_ = 72, *n*_daf_ = 17). Additionally, age‐specific reproduction and individual fitness are shown following developmental *daf‐2* knockdown for individuals maintained at (E, F) 15°C (*n*_ev_ = 74, *n*_daf_ = 64), or (G, H) 25°C (*n*_ev_ = 54, *n*_daf_ = 58), or (I, J) *daf‐2* knockdown during adulthood at 20°C (*n*_ev_ = 95, *n*_daf_ = 85). Individuals used in these assays were from an entire cohort of ~100 eggs selected before hatching, and 24‐h daily periods were measured from the time the first individual was sexually mature. (K) Age‐specific reproduction and (L) individual fitness following parental and offspring treatments of *daf‐2* RNAi or ev (*n* > 40 for all treatments). These individuals were obtained from a cohort of ~50 eggs.

### 2.4 IIS Downregulation Carries Costs for Developmental Arrest Entry and Exit

Offspring of parents that experienced *daf‐2* RNAi from hatching entered dauer more frequently in crowded conditions ($\chi_{1}^{2}$ = 25.597, *p* < 0.0001, Figure 5A,B, Table S14). Interestingly, individuals also entered dauer more frequently in populations with higher concentrations of bacterial food (*p* = 0.005; Table S14, Figure 5B). This is likely due to the higher population density under higher food concentrations (*F*_1,17_ = 13.792, *p* = 0.002; Table S15), which likely also explains the presence of dauer entry in some controls. When *daf‐2* knockdown occurred during L1 arrest recovery, individuals with *daf‐2* knocked down from recovery onwards grew to a larger size than controls ($\chi_{3}^{2}$ = 8.324, *p* = 0.040, Figure 5C), but grew at the same rate as controls ($\chi_{3}^{2}$ = 0.648, *p* = 0.885, Figure 5D, Table S16). Additionally, developmental *daf‐2* knockdown that occurred only during development or from development onwards after L1 recovery took longer to begin producing eggs ($\chi_{3}^{2}$ = 540.59, *p* < 0.0001, Figure 5E, Table S17). These results suggest that parental developmental *daf‐2* knockdown impairs environmental sensing in their offspring, while larval *daf‐2* knockdown in larval nematodes also slows recovery time from developmental arrest.

**FIGURE 5:**
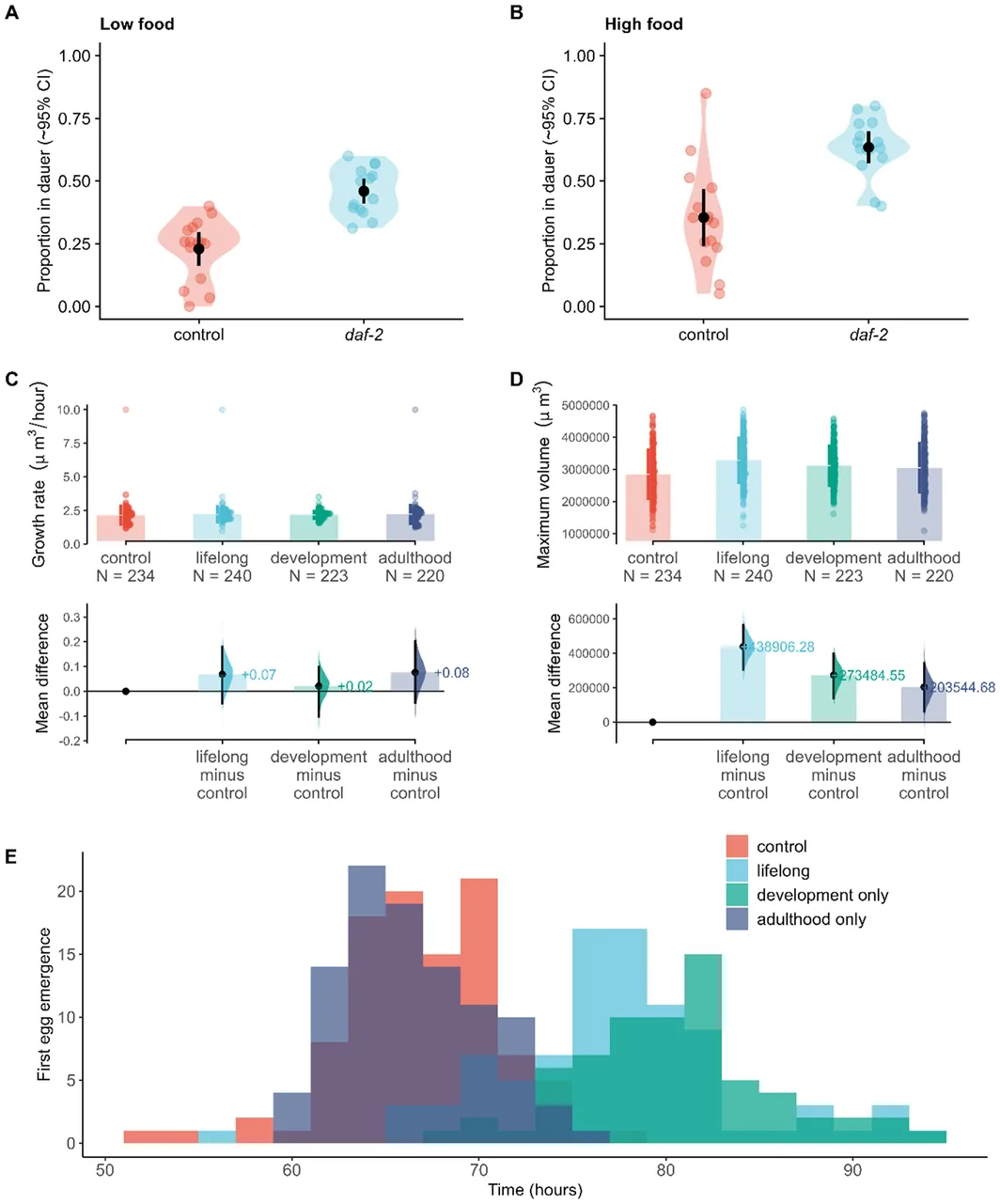
IIS downregulation carries costs during development that are environment‐dependent: Dauer entry rates in offspring produced from parents exposed to developmental *daf‐2* RNAi following offspring exposure to (A) limited or (B) *ad libitum* food densities. The proportion of dauers from each of 3 technical replicate samples from each population (*n* = 15 populations per RNAi treatment per food regime) is shown. (C) Growth rate, (D) maximum volume, and (E) time at first egg production after *daf‐2* RNAi mediated knockdown during development only (*n* = 223), adulthood only (*n* = 220), or lifelong (*n* = 240) compared to controls (ev; *n* = 234) following L1 arrest. Growth rate and maximum volume (C, D) are represented by mean differences from bootstrapped estimations.

### 2.5 Adulthood IIS Downregulation Does Not Slow Population Growth in Semi‐Natural Conditions

Following developmental *daf‐2* RNAi treatment, we found a significant treatment by day by block interaction effect on population growth in semi‐natural conditions ($\chi_{3}^{2}$ = 21.818, *p* = < 0.0001, Figure 6A, Table S18), suggesting that *daf‐2* RNAi populations grew faster than control populations in some environmental conditions but slower in other environmental conditions (Figure S1, Table S19). When *daf‐2* RNAi was applied during development and 24‐h of early adulthood, there was no evidence that treatment affected population growth (treatment: $\chi_{1}^{2}$ = 0.116, *p* = 0.940, Table S20, Figure 6B) but rather was dependent on environmental conditions (Day*Block: $\chi_{1}^{2}$ = 14.460, *p* < 0.0001, Figure S2, Tables S20 and S21). For populations founded from individuals after 24‐h adulthood *daf‐2* RNAi, there was no evidence that populations grew faster or slower than controls (Treatment*Day: $\chi_{3}^{2}$ = 0.047, *p* = 0.828; Figure 6C, Figure S3, Table S22). See Figure S4 for daily average ambient air temperature for each block.

**FIGURE 6:**
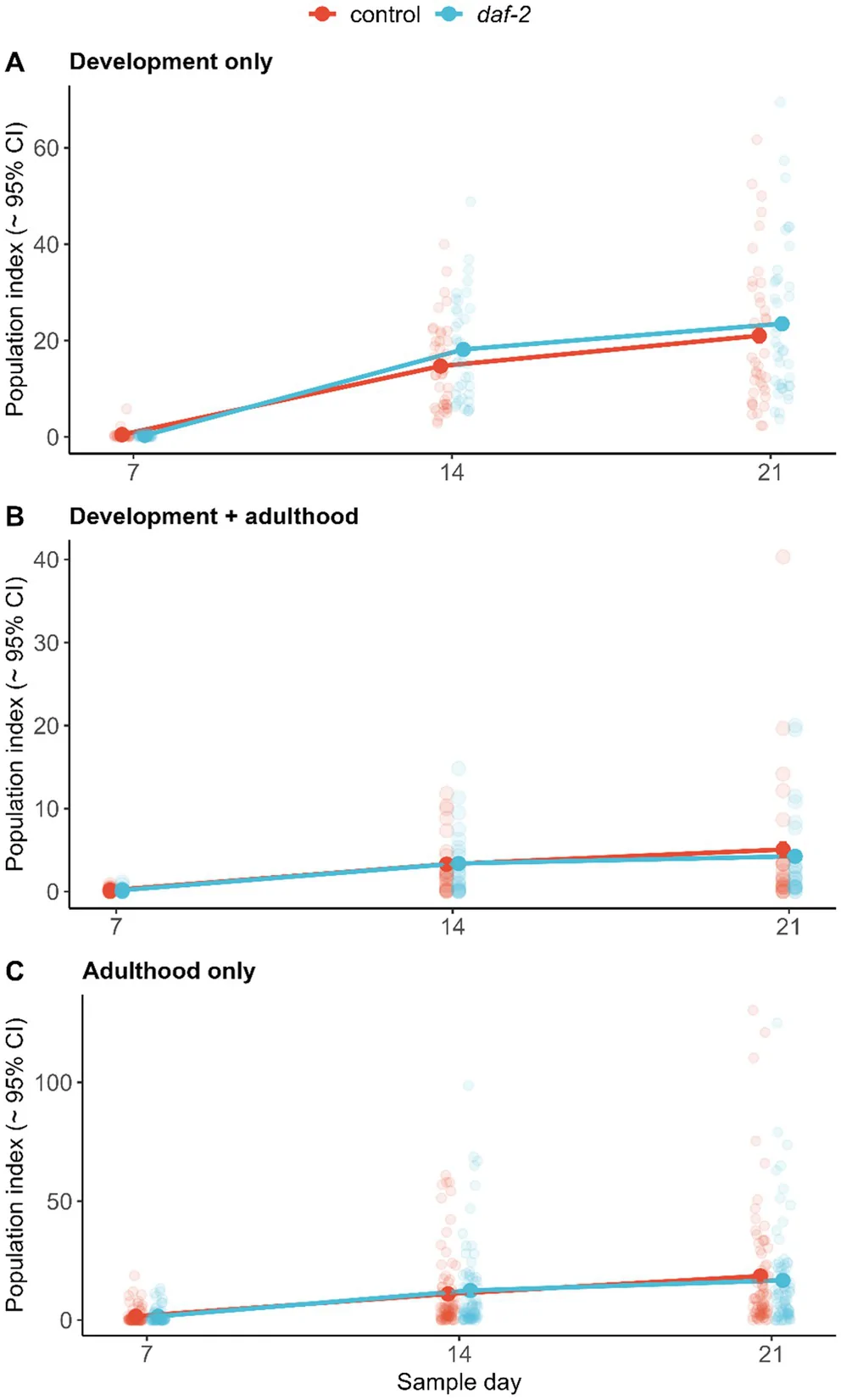
IIS downregulation during adulthood carries no cost to population growth: Population index measured as the number of individuals in a 5‐mL soil sample from a 250‐mL microcosm each containing a nematode population founded by 50 individuals following control empty vector feeding or either (A) developmental *daf‐2* RNAi (*n* = 39 populations per treatment), (B) developmental and 24‐h Day 1 adulthood *daf‐2* RNAi (*n* = 24 populations per treatment), or (C) 24‐h *daf‐2* RNAi from sexual maturation (*n* = 39 populations per treatment). Populations were sampled weekly over a 21‐day period.

## 3 Discussion

IIS regulates environmental sensing and integrates diverse environmental cues into downstream physiological processes that regulate organismal life‐history. Our results overturn the prevailing paradigm that reduced IIS during development lowers fitness in *C. elegans* through slowed down development and delayed reproduction. Instead, reduced IIS during development can be beneficial for development time, early‐life reproduction, lifetime reproductive success, individual fitness, and survival. However, this comes at the cost of inadequate environmental sensing leading to overly frequent entry into a non‐growing and non‐reproducing developmental diapause and reproductive costs associated with recovery from arrest phases.

It has been well‐established that lifelong *daf‐2* RNAi approximately doubles lifespan in *C. elegans* (Dillin et al. 2002; Lind et al. 2019). Here, we show that even a brief, transient treatment of *daf‐2* RNAi during development or adulthood still increases lifespan substantially, although increased temperatures ameliorate the lifespan extension provided by developmental *daf‐2* knockdown. Evidently, lifespan‐extending effects are due to persistent downstream *daf‐2* effects, as the affected phenotype is preserved long after *daf‐2* expression levels return to that of wildtype controls. This is likely due to the numerous downstream genes regulated by the IIS pathway that are implicated in stress response, antimicrobial resistance, and protein degradation (Murphy et al. 2003), and the known benefits of reduced IIS in adulthood to increased proteostasis (Taylor and Dillin 2011), stress resistance (Carlsson et al. 2021), anti‐bacterial (Fabian et al. 2021), and anti‐viral immunity (Duxbury et al. 2024). These effects may be preserved long after experimental IIS downregulation ends.

Contrary to previous work, we found that developmental *daf‐2* downregulation can improve all measured aspects of reproduction, including early reproduction and total reproductive output. Our finding that both LRS and individual fitness improved following developmental *daf‐2* knockdown suggests that the benefits arise from both accelerated development and enhanced reproductive capacity in the fastest developers. Due to our experimental design, we included all individuals from each cohort, including the fastest and the slowest developers. Typical experimental designs are based on setting up reproductive assays from the time when at least 50% to 75% of individuals have sexually matured to make it easier to collect large numbers of similarly aged individuals. However, this could lead to the earliest developers having already surpassed the desired larval stage. Differences in developmental time between treatments can thus lead to inadvertent ‘selective appearance’ that can bias the results. Our results suggest that those earliest developers benefit not only from initiating reproduction earlier but also increased reproductive capacity. Our re‐analysis confirms this by showing that only including individuals that reach sexual maturity on or after the peak maturation time for controls results in lower early‐life fecundity and overall fitness as previously reported in previous studies. This is because early‐maturing *daf‐2* RNAi animals that start reproducing earlier than controls are missed when using this approach. The resultant 10% increase in LRS we found is in line with what would be expected to carry consequences in nature (Bouwhuis et al. 2012). However, this beneficial effect was dependent on temperature, as it was lost when temperatures were lower or higher than the standard laboratory temperature, though no cost was observed either.

These findings of improved fitness under developmental IIS downregulation are not predicted by the DTA, nor by the broader evolutionary theory of ageing, which maintains that strong selection in early life should optimise development, growth, and reproduction. Although we saw no direct cost to reproductive output or timing, we found that reduced insulin signalling impairs environmental sensing during larval development. This is critical for *C. elegans*, as larvae may perceive environmental stressors as greater than they are and enter dauer unnecessarily. If this produces a long enough delay to reproduction, this could effectively reduce an individual's fitness to zero compared to non‐arrested counterparts. Slowed recovery from developmental arrest due to reduced IIS levels will also cause reproductive delays. This discrepancy between accelerated development following developmental *daf‐2* knockdown and delayed egg production following arrest recovery can be explained by the dual role of IIS in both regulating developmental pace and integrating environmental cues. During active larval development, reduced IIS accelerates progression through larval stages. However, when IIS is reduced during or after recovery from developmental arrest, the impaired environmental sensing delays the appropriate transition back into reproductive development, resulting in a delayed onset of egg production despite a faster intrinsic developmental pace.

The mechanisms by which developmental IIS influences environmental sensing and developmental plasticity likely involve the integration of multiple environmental cues through ILPs. Several ILPs regulate the dauer decision by modulating DAF‐2 signalling, with some acting antagonistically to promote or inhibit dauer entry (Hung et al. 2014; Cornils et al. 2011; Li et al. 2003). Developmental *daf‐2* knockdown effectively mimics the signal of reduced ILP production that normally occurs under stressful conditions, biassing the dauer decision toward entry even when environmental conditions do not warrant it. Additionally, IIS interacts with the TGF‐β and steroid hormone pathways that jointly regulate dauer decision‐making, such that reduced IIS during development may disrupt the coordinated integration of these pathways (Murphy and Hu 2018). Our finding that developmental daf‐2 knockdown also slows L1 arrest recovery further suggests that IIS is required not only for the initial dauer decision but also for the appropriate sensing of environmental improvement that triggers recovery from arrest. These mechanisms provide a proximate explanation for why strong selection during development would maintain IIS at levels that optimise environmental sensing, even if modest fitness benefits could be gained from reduced IIS in stable environments.

This highlights that the timing of IIS downregulation relative to the developmental stage and environmental context critically determines whether its effects on development are beneficial or costly. In nature, the boom‐and‐bust population dynamics experienced by *C. elegans* require entering arrest stages with appropriate timing to survive harsh conditions and allow migration to a new food source (Schulenburg and Félix 2017). This suggests that larval environmental sensing and response would be under strong selection, preventing changes in IIS regulation that may produce modest fitness improvements in moderate environmental conditions.

Because laboratory findings may not always translate into similar results in ecologically relevant environments, we tested the effects of age‐specific transient downregulation of IIS on population growth rate in semi‐natural conditions. Developmental *daf‐2* RNAi in founder populations resulted in significant effects on population growth rate over several generations, while the direction of the effects differed strongly across blocks that were conducted in different environmental conditions. In some blocks, populations started by *daf‐2* RNAi parents developed faster and reached large population size as would be expected based on the laboratory results. In other blocks, there were no overall effects, or the growth rate of *daf‐2* RNAi populations was slower, suggesting that environmental factors may have other effects on population growth rate that were unaccounted for in the laboratory environment. On the other hand, adulthood‐only *daf‐2* RNAi, which increases stress resistance and immunity in the laboratory tests, did not translate into effects on population growth rate. This latter result is in line with an earlier study simulating fluctuating temperature and light stress conditions in the laboratory microcosm (Carlsson et al. 2021).

What kind of environmental effects could contribute to variation in population growth rates in response to *daf‐2* RNAi across blocks? We found that parental *daf‐2* RNAi impairs environmental sensing and thus dauer regulation in their offspring, causing higher than expected rates of dauer entry when it may not be necessary. We hypothesise that blocks where parental *daf‐2* RNAi populations developed slower and reached lower population sizes than controls may have experienced more stressful environmental conditions that led to a higher proportion of young animals entering reproductive diapause, while their counterparts in control populations continued to develop and reproduce. These changes could include changes in temperature, moisture content, and bacterial community composition, as well as the introduction of potential predators or pathogens from the natural environment. In a competitive natural setting, this would have major fitness consequences, providing insight as to why selection would not act to reduce IIS in early life. Additionally, our experimental paradigm could even have reduced the negative effect of developmental *daf‐2* in an outdoor setting because the offspring of well‐fed worms are more likely to enter dauer (Harvey and Orbidans 2011), and our parental generation was well‐fed in the laboratory increasing the chance that control worms will enter dauer thereby reducing differences between treatments.

Overall, these results suggest that natural selection acts on insulin signalling that regulates environmental sensing during development, and downregulation of this pathway in parents results in increased probability that their offspring will enter reproductive diapause instead of development and reproduction. Because IIS pathway is a life‐history nexus that regulates development, growth, reproduction and lifespan, and because downregulation of IIS in development accelerates development, improves reproduction and increases longevity, there is a genetic trade‐off, where the complexity of IIS acts as a biological constraint on adaptive life‐history evolution (Cohen et al. 2020). While downregulation of IIS in adulthood could, in theory, be beneficial as it avoids the developmental constraints, there is currently no evidence that there is selection on post‐developmental function of IIS in natural environments. Our results suggest that while adulthood‐only downregulation of IIS increases lifespan, it does not increase population growth rate. Developmental downregulation of IIS does increase population growth rate, but only in some environmental conditions and at the cost of reduced environmental sensing. We suggest that such genetic trade‐offs, where selection optimises complex molecular signalling networks for early‐life performance from zygote to age at first reproduction, at the cost of late‐life performance, can be a common cause underlying the evolution of ageing.

## 4 Methods

### 4.1 Experimental Model

#### 4.1.1 Strain

The N2 (Bristol) wild‐type strain of *Caenorhabditis elegans* nematodes obtained from the *Caenorhabditis* Genetics Centre, which is funded by the NIH Office of Research Infrastructure Programs (P40 OD010440), was used throughout this experiment. Nematodes were thawed from −70°C then bleached using a solution of NaOCl and NaOH to obtain a population free of bacterial and fungal contaminants. The populations were maintained in climate chambers (20°C) for a minimum of two generations prior to any experiments on 90 mm Nematode Growth Medium (NGM) plates containing 10 mg L^−1^ of Nystatin and 100 mg L^−1^ of ampicillin to prevent infection. They were fed *Escherichia coli* OP50‐1 (pUC4 K), obtained from J. Ewbank at the Centre d'Immunologie de Marseille‐Luminy, France. Timed egg‐lays were used to produce age‐synchronised populations.

#### 4.1.2 RNAi

To induce *daf‐2* RNAi knockdown, nematodes were fed *E. coli* HT115 (DE3) that contained a pL4440 vector which contained an insert that expressed dsRNA which corresponds to the *daf‐2* gene in *C. elegans*. Control nematodes were fed the same *E. coli* clone containing the pL4440 vector that did not carry an insert (hereon, empty vector (EV)). The clone cultures were seeded onto NGM plates containing 10 mg L^−1^ of nystatin and 50 mg L^−1^ ampicillin, as well as 1 mM IPTG for transcription induction of the RNAi operon. All assays were blinded with respect to treatment.

### 4.2 Method Details

#### 4.2.1 qRT‐PCR

To identify how *daf‐2* downregulation changes following transient adulthood *daf‐2* RNAi exposure, we used quantitative reverse transcriptase polymerase chain reaction (qRT‐PCR). Treatment began at late L4 larval stage and ended 24 h later. Nematodes used for this assay were maintained under standard conditions described previously. Individuals were then collected for RT‐qPCR, every 12 h following removal from treatment up to 48 h. Nematodes were first placed on a clean agar plate to allow ingested bacteria to be excreted (Ghafouri and McGhee 2007) and to remove surface bacteria (Chauve et al. 2020). Individuals were lysed in lysis buffer (1:100 diluted DNase; Ambion Power SYBR Green Cells‐to‐Ct kit), then centrifuged down into the bottom of the tubes to be flash frozen with liquid nitrogen (Chauve et al. 2020). To release nucleic acids, we performed 10 freeze–thaw cycles, by cycling the PCR tubes between liquid nitrogen and a 40°C water bath, before placing in a thermal mixer to homogenise (Eppendorf ThermoMixer C) set at 4°C for 30 min at 1800 rpm (Chauve et al. 2020).

Following the manufacturer protocol from the Ambion Power SYBR Green Cells‐to‐Ct kit, we reverse transcribed the RNA. We ran the qRT‐PCR on an Applied Biosystems 7500 Real‐Time PCR System with a PCR cycle of: 10 min at 95°C, then 40 cycles of 15 s at 95°C and 60 s at 60°C with a total reaction volume of 20 μL containing 4 μL cDNA. We used primers specific for the target gene of interest (*daf‐2*) and for the reference gene, *actin‐3* (T04C12.4) (Akay et al. 2017). Primers for *daf‐2* (Chauve et al. 2020) and *actin‐3* (Akay et al. 2017) were designed based on MIQE guidelines (Bustin et al. 2009). Primer sequences are listed in Table S24.

#### 4.2.2 Fitness and Lifespan Assays in the Laboratory

First, the efficacy of brief, transient *daf‐2* RNAi exposure was tested in a laboratory setting to validate its later use in the outdoor assays. If this brief treatment is effective at extending lifespan, this opens the door for a wider array of future experiments that will not depend on continuous exposure to this specific bacterial food. The population used for these assays was produced by age‐synchronised adult nematodes at their reproductive peak. These were then exposed to either lifelong ev or *daf‐2* RNAi, or a 24‐h treatment of *daf‐2* RNAi during L1 stage or on day 1 of adulthood. Individuals were kept on 35 mm agar plates in groups of ten and transferred to new plates every other day. Daily observations of response to touch were used to determine survival. Using the same assay methods, the effects of *daf‐2* RNAi knockdown from hatching until sexual maturity on lifespan were tested at 15°C, 20°C, and 25°C. These temperatures were used because they are common laboratory maintenance temperatures for *C. elegans* with significant effects on life‐history timing (Stiernagle 2006; Zhang et al. 2015).

To determine fitness effects of *daf‐2* downregulation during development and adulthood, larvae were maintained in groups until they reached sexual maturation, when they were transferred onto individual plates. The developmental *daf‐2* treatment consisted of nematodes on *daf‐2* RNAi food from egg stage until late L4 kept at 15°C, 20°C, or 25°C. Time (hours) to sexual maturity (identified by vulva eversion) was measured to assess whether *daf‐2* RNAi treatment affected time to initiation of reproduction. This is critical because early offspring output plays a key role in genetic contribution in growing populations (de Vries et al. 2023). Additionally, nematodes were kept in densities of 5 or 50 to identify any effects of population density on development time. Individuals were transferred every 24 h, with the 24‐h clock beginning from the time the first individual reached sexual maturity to test for any effects of developmental time on reproduction. The adulthood *daf‐2* treatment began at late L4 for a 24‐h period at the standard maintenance temperature (20°C).

Development time and reproduction were also assessed for multigenerational *daf‐2* knockdown effects, with a full‐factorial design for *daf‐2* and ev treatments for parents (P0) and offspring (F1). These assays were performed as another way to test for the effects of differences in laboratory methodologies, as sometimes parents may be treated in addition to offspring. If parent and offspring *daf‐2* RNAi treatments interact, this could further explain disparities in results between laboratories. Parents were given an adulthood treatment of *daf‐2* RNAi or EV through reproduction starting in adulthood. This was performed in two experiments across two laboratories to test the robustness of any effects observed and to ensure that effects observed are not solely due to laboratory‐ or population‐specific effects. See Table 1 for a direct comparison of the experiments. In Experiment 1, development time was assessed as described above. In Experiment 2, for measurement of the effect of *daf*‐*2* RNAi on developmental duration by time lapse live imaging, individual eggs were deposited in agarose‐based chambers (4.5% agarose dissolved in S‐basal), as described by Stojanovski et al. (2023) and imaged every 20 min over the course of their entire development. Chamber dimension was 600 × 600 × 20 μm. Bacterial strain HT115 containing plasmids for expression of *daf‐2* RNAi was used as food source in chambers. The same strain carrying an empty plasmid (L4440) served as the negative control. RNAi bacteria were prepared by seeding saturated overnight cultures onto IPTG plates and left to dry at 37°C. RNAi bacteria were scraped off from IPTG plates and filled into agarose chambers. Imaging was performed on Squid microscopes from Cephla inc (https://cephla.com/), equipped with a Blackfly S CMOS camera (BFS‐U3‐63S4M‐C, Teledyne vision solutions) and a 10×/N.A. 0.4 Olympus objective (Li et al. 2020). Segmentation of worms from the background was conducted using a UNET++ based deep learning model embedded in a custom‐made Python script. The detection of hatch times and moults was conducted from volume trajectories as previously described by Stojanovski et al. (2022).

**TABLE 1:** Comparison of Experiments 1 and 2 to test the combined effects of parental adulthood *daf‐2* knockdown and offspring developmental *daf‐2* knockdown on development time.

	Experiment 1	Experiment 2
Housing	35‐mm NGM agar plates containing IPTG in groups of up to 50 individuals	600 × 600 × 20 μm chamber containing 4.5% agarose dissolved in S‐basal, housed individually
Measurement of maturation	Vulva eversion	Larval growth to identify M4 moult
Measurement method	Visual observation of vulva development performed hourly	Imaging (every 20 min) to measure larvae, using growth resumption after lethargus to indicate moult
Median time to maturation for control	60 h	40 h

#### 4.2.3 Dauer Assay

Assays to assess whether offspring produced following developmental *daf‐2* RNAi knockdown experienced dauer entry in inappropriate conditions were performed to identify costs to environmental sensing. Age synchronised populations were produced by worms at their peak reproductive period, laying eggs for 4 h onto 90 mm agar plates seeded with either bacteria expressing dsRNA against *daf‐2* or an empty vector as in previous assays. Offspring were left to mature on their respective RNAi treatment. At day two of adulthood, worms were removed from plates by rinsing with S‐buffer, leaving only the eggs on the plates. The number of eggs obtained was over 180,000. Plates were then bleached and rinsed twice with S‐buffer to remove any traces of bleach. The number of eggs in the S‐buffer was estimated by sampling small volumes onto agar plates and counting them under the microscope, whereafter the volume required to contain 9000 eggs was placed into 5 mL tubes. Bacteria were added according to two food regimes, high (38 mg) and medium (19 mg). S‐buffer was added to make up a final volume of 1 mL. To acquire bacterial broth with the desired concentrations, *E. coli* was first grown in Terrific Broth (TB) containing 100 μg mL^−1^ Ampicillin at 37°C for 20 h. The *E. coli* was then concentrated using centrifugation to 76 mg mL^−1^ diluted in S‐buffer containing 100 μg mL^−1^ ampicillin and nystatin and 12.5 μg mL^−1^ tetracycline to stop bacterial growth.

Using these methods, the aim was to produce crowded conditions at different food concentrations, as these conditions can promote dauer formation if larvae perceive the conditions as poor enough to prevent successful reproduction. Populations were left in liquid medium for 5 days on a rocker in 20°C. After 5 days, the number of dauers in populations was estimated by sampling 5 μL of the liquid and pipetting onto a 35‐mm agar plate to count. Three technical replicates were used to measure the number of dauers in 5 populations per RNAi and food treatment combination (*n* = 20 populations in total).

#### 4.2.4 L1 Arrest Recovery Assays

The effects of development only, lifelong, and adulthood only *daf‐2* RNAi knockdown were tested against controls (ev) on growth and reproduction following L1 arrest. If reduced developmental IIS has harmful effects on environmental sensing, then *daf‐2* RNAi treated larvae would be expected to experience delayed recovery, thus reducing fitness. This assay was performed using high throughput Development and Reproductive Toxicology (DART) assays with the Nagi Biosciences SydLab One platform. Nematodes were maintained on NGM agar plates until adults were collected and placed in S‐medium to lay eggs. L1 arrest was used to age‐synchronise populations for a duration of no more than 12 h. These L1 nematodes were injected into the SydLab One platform and monitored over 4 days via images to measure growth and reproductive output.

#### 4.2.5 Outdoor Setup and Population Sampling

While laboratory tests have successfully shown that IIS downregulation via *daf‐2* RNAi knockdown in adulthood extends lifespan without costs (Lind et al. 2019), little is known about how IIS downregulation affects natural populations. To test the effects of *daf‐2* RNAi knockdown in the wild, founder populations were cultivated in the laboratory, exposed to *daf‐2* RNAi, and released into mesocosms that were placed outdoors in a forest of the University of East Anglia campus in Norwich, UK. To create the most natural environment for the nematodes that could reasonably be repeatedly sampled, an organic, peat‐free compost was used as a medium. Each *C. elegans* population was housed in a 250‐mL conical tube filled with compost. Holes were drilled into the bottoms of the tubes to allow for drainage of excess liquid in the event of heavy rain. The compost was autoclaved no more than 7 days prior to use to ensure that any nematodes already present in the compost were killed. The biotic environment was re‐introduced through a mixture of ten bacterial strains (Table S25) found to be associated with *C. elegans* in natural environments (Dirksen et al. 2020). The bacterial strains were individually maintained in LB broth containing no additives and kept at 4°C after growing (see Table S25 for growth requirements). The bacterial strains were then mixed in equal volumes and 60 mL (the amount required to fully saturate 250 mL of compost) of this mixture was added to each compost tube and left for 48 h at room temperature to grow before nematode populations were added.

Once outdoors, the tubes were suspended above a heavily salted water bath in racks to prevent environmental contamination, as any nematodes that escaped from the drainage holes would be killed by osmotic stress. The setup was protected by a wire mesh cage that was staked to the ground to prevent any disruption from wild animals.

To observe the effects of developmental *daf‐2* RNAi treatment on population growth in a natural environment, eggs were picked onto either *daf‐2* RNAi or EV bacterial plates and remained there until the late L4 stage (*n* = 39 populations per treatment). The same was done for the development + adulthood *daf‐2* RNAi treatment, except worms were left on their treatment until Day 2 of adulthood (*n* = 24 populations per treatment). For the effects of transient adulthood downregulation of IIS on population growth, populations were founded by nematodes following either 24‐h of *daf‐2* RNAi or EV bacteria exposure from late L4 (*n* = 79 *daf‐2* populations, *n* = 78 EV populations). Performing this treatment during early adulthood only ensured that the majority of the nematodes' reproductive period was spent in semi‐natural conditions, including their peak of reproduction. At the end of their treatment, nematodes were placed into populations of 50 onto clean, unseeded 35‐mm agar plates. They were then rinsed into a tube of compost using 3 mL of the bacterial cocktail described above. Once outdoors, the populations were fed with 25 mL of the bacterial mixture twice weekly (every 3–4 days).

Populations were sampled on days 7, 14, and 21 to create a population index for each day to estimate population growth over time. As we were unable to track individuals in this setup, this allowed comparison of population growth rate as a fitness proxy across treatments to identify any costs at the population level from IIS knockdown and to identify whether IIS downregulation is an evolutionarily relevant mechanism in nature. To do this, a 5‐mL compost sample was taken from the top of the tube following mixture of the top half of the compost. The bottom of the tubes retained liquid from feeding and precipitation and was avoided. The samples were placed in 15‐mL conical tubes, then taken back to the laboratory to extract the nematodes. Each sample was first cleaned with reverse osmosis purified (RO) water to remove smaller particles by filling the tube to a total volume of 10 mL, then dissolving the compost as completely as possible and centrifuging at 1800 g for 5 min. The nematodes were then extracted using centrifugal flotation. Each tube was filled up to 10 mL with MgSO_4_ solution with a specific density of 1.15 and thoroughly mixed to dissolve all compost, then centrifuged at 1800 g for 5 min. The supernatant was gently mixed, and 0.1 mL of this liquid was pipetted onto 6 35‐mm agar plates to obtain 6 technical replicates per population. The plates were allowed to dry, and the extracted nematodes were counted on the same day.

The outdoor assay was run over 5 blocks for the adulthood *daf‐2* treatment in the summer and autumn in 2022 and summer in 2023. The two final blocks of the adulthood assay included a developmental *daf‐2* plus early adulthood *daf‐2* RNAi treatment. The developmental *daf‐2* treatment assays were performed in summer and autumn in 2022 and 2023 across four blocks. Exact dates for each block can be found in Figure S2 (developmental *daf‐2* treatment), Figure S3 (developmental + adulthood *daf‐2* treatment) and Figure S4.

### 4.3 Statistical Analysis

All analysis, exploration and visualisation of data was performed using R v. 4.2.0 (R Core Team 2022). Model diagnostics were assessed by simulating residuals and tested for zero‐inflation and overdispersion using the DHARMa package v. 0.4.6 (Hartig and Lohse 2022). Where necessary, an observation level random effect was included to prevent overdispersion. Models were reduced by removing factors and/or interactions with non‐significant effects (*p* < 0.05). Data were visualised using ggplot v. 3.4.4 (Wickham 2016) unless otherwise stated.

For survival analyses, individuals whose death was due to matricide were excluded (see Tables S1–S4 for analyses with matricides included). The data did not always conform to the assumptions of a Cox proportional hazards model (cox.zph test, GLOBAL term: *p* < 0.05). In these cases, an event history analysis with a binomial GLMM from the glmmTMB package v. 1.1.9 (Brooks et al. 2017) was used. Where results were consistent with those of the Cox models, the results from the Cox models were used to simplify comparisons of model interpretation across experiments.

Relative gene expression was calculated as the mean ΔCt (Ct_daf‐2_–Ct_actin_) across the two technical replicates per sample. The fold change in gene expression (2^−ΔΔCt^) was calculated per RNAi treatment using the mean across biological samples (Schmittgen and Livak 2008). Relative gene expression was analysed using a linear mixed effects model with Gaussian error structure to determine the effect of *daf‐2* RNAi treatment over time. qRT‐PCR Plate number was fitted as a random intercept, which accounted for the effect of the day the plate was run, as plates were run on individual days. Individual experimenter and location on plate were not taken into account in models as all qPCR assays were performed by the same person, and placement of samples across treatments and times within plates was randomised.

Time to sexual maturity was always positively skewed, and due to the continuous nature of our predictor a generalised linear model with a Gamma distribution and log link function, with offspring treatment (and parental treatment, when necessary) as fixed factors. Three measures of fitness were examined in this study: age‐specific reproduction, lifetime reproductive success (LRS), and individual rate‐sensitive fitness (λ_ind_). Age‐specific reproduction measures allow us to identify how reproductive schedules changed with treatment, and individual fitness specifically models how treatment impacts early reproduction, which is critical to understanding fitness in populations with rapid or exponential growth. LRS was used to test the reproductive capacity, which allowed us to identify whether changes in timing might impact total reproductive output. To analyse the effects of developmental and adulthood *daf‐2* treatments on age‐specific reproduction, zero‐inflated negative binomial (ZI‐NB) models were used, with Treatment, Day, and Day^2^ as fixed effects. Day^2^ was included to account for the quadratic distribution of number of offspring produced over time. Day was included as a zero‐inflated formula predictor to improve model fit due to the high number of zeroes observed in the earliest and latest stages of the reproductive schedules, and ID was fitted as a random intercept to account for repeated measures of individuals over time. For multigenerational treatment analyses, Parent Treatment was also included as a fixed factor.

LRS was calculated as the total number of offspring produced by an individual over the assessment period. To calculate individual fitness (λ_ind_), the *lambda*() function from the popbio package v. 2.7 (Stubben and Milligan 2007) was used to construct Leslie matrices (Leslie 1945) which were used to calculate the dominant eigenvalue. Two days were added to the fertility schedule to represent development time (from egg until maturity). As all individuals typically reproduce in these laboratory populations, LRS and individual fitness data were normally distributed. Both LRS and individual fitness were analysed using a linear model (LM) with a Gaussian error distribution and a fixed factor of Treatment.

Dauer formation rates were analysed using a binomial GLMM to model the proportion of individuals entering dauer while accounting for variation in the total number of individuals scored in each replicate. The response was specified as the numbers of dauer and non‐dauer individuals using cbind (dauer, non‐dauer) with treatment and food amount as fixed factors, with technical replicate included as a random intercept nested within population. The effects of treatment and food concentration on population size for this assay were analysed using a LM. For the effect of treatment on growth rates, maximum volume and time at first egg production following L1 recovery, linear mixed effects models with a Gaussian error distribution were used to include ‘Chamber’ (indicator of location in microchips used in SydLab One assay) as a random effect to account for any effects of location on the microchip.

Population growth data from outdoor populations were generally overdispersed and zero‐inflated across sample days and blocks. To analyse them, we used GLMMs with a negative binomial distribution, including Block and/or Day as zero‐inflation factors and Block as a dispersion factor where appropriate, comparing model fits across variations using the lowest Akaike Information Criterion (AIC) value to select the best model. This was done with caution when problems with dispersion persisted, as this can inflate AIC values, and in these cases model diagnostics were used alongside these values. Plate ID explicitly nested within Population ID was included as a random factor to account for resampling of populations over time points to include an observation level random effect and to account for repeated sampling across time of populations. Block was included as a fixed factor due to having few of them and the strong effect they had on population size over time. Individual block data were also analysed using GLMMs with negative binomial distributions with Day included as a zero‐inflation factor. Due to the smaller sample size within blocks, when necessary, glmmTMBcontrol optimisers were used to allow model convergence. See Tables S19, S21, and S23 for details on models for individual blocks.

## Author Contributions

Conceptualization: S.D.I., S.I., A.A.M. Formal analysis: S.D.I., P.L. Funding acquisition: B.T., S.I., A.A.M. Investigation: S.D.I., A.K., P.L., H.C., A.B. Methodology: S.D.I., P.L., B.T., A.A.M. Supervision: B.T., S.I., A.A.M. Visualisation: S.D.I. Writing – original draft: S.D.I., A.A.M. Writing – review and editing: S.D.I., A.K., P.L., B.T., S.I., A.A.M.

## Funding

This work was supported by the European Research Council, SELECTHAPLOID 101001341. Natural Environment Research Council, NE/W001020/1. European Cooperation in Science and Technology, IZCO‐0‐240511.

## Conflicts of Interest

The authors declare no conflicts of interest.

## Supporting information


**Table S1:** Event history analysis with binomial GLMM for survival in nematodes treated with transient *daf‐2* knockdown either during development, D1 of adulthood, or entire life with and without matricides censored.
**Table S2:** Cox proportional hazards analysis for survival in nematodes treated with daf‐2 for the duration of development in 20°C with and without matricides censored.
**Table S3:** Cox proportional hazards analysis for survival in nematodes treated with daf‐2 for the duration of development in 15°C with and without matricides censored.
**Table S4:** Cox proportional hazards analysis for survival in nematodes treated with *daf‐2* for the duration of development in 25°C with and without matricides censored.
**Table S5:** Anova results and estimated marginal means contrasts from linear model of the effect of RNAi treatment and time on normalised relative gene expression of *daf‐2* in *C. elegans* up to 48 h post removal from *daf‐2* RNAi bacterial food.
**Table S6:** GLM for Gamma distribution with log link analysis of time to sexual maturity in nematodes after exposure to *daf‐2* RNAi throughout development in 15°C, 20°C, and 25°C.
**Table S7:** Model summary and Anova from a Gamma log link family model to analyse the effect of parental adulthood *daf‐2* knockdown combined with offspring *daf‐2* knockdown on developmental timing.
**Table S8:** Model summary and Anova output from analysis of age‐specific reproduction for developmental daf‐2 in 20°C using zero‐inflation negative binomial GLMM. Model summary and Anova output from analysis of LRS and individual fitness from a general linear model.
**Table S9:** Model summary and Anova output from analysis of age‐specific reproduction following developmental daf‐2 knockdown measured only in individuals that matured on or after the peak of maturation for controls using zero‐inflation negative binomial GLMM. Model summary and Anova output from analysis of LRS and individual fitness from a general linear model.
**Table S10:** Model summary and Anova output from analysis of age‐specific reproduction for developmental daf‐2 in 15°C using zero‐inflation negative binomial GLMM. Model summary and Anova output from analysis of LRS and individual fitness from a general linear model.
**Table S11:** Model summary and Anova output from analysis of age‐specific reproduction for developmental daf‐2 in 25°C using zero‐inflation negative binomial GLMM. Model summary and Anova output from analysis of LRS and individual fitness from a general linear model.
**Table S12:** Model summary and Anova output from analysis of age‐specific reproduction for adulthood only *daf‐2* in 20°C using zero‐inflation negative binomial GLMM. Model summary and Anova output from analysis of LRS and individual fitness from a general linear model.
**Table S13:** Model summary and Anova output from analysis of age‐specific reproduction following parental adulthood *daf‐2* knockdown and offspring developmental *daf‐2* knockdown using zero‐inflation negative binomial GLMM. Model summary and Anova output from analysis of LRS and individual fitness from a general linear model.
**Table S14:** Proportion of dauers produced by parents with daf‐2 knockdown from egg stage to early adulthood and EV control parents in high and medium food concentrations analysed using a binomial GLMM using glmer() with the cbind() function with number of individuals in dauer and number of individuals not in dauer as successes and failures, respectively.
**Table S15:** Estimated relative population size in vials containing populations founded by parents that experienced *daf‐2* knockdown from egg stage to early adulthood and control parents in high and medium food concentrations analysed using GLM with Gaussian error structure.
**Table S16:** Analysis of growth rates (measured at half of maximum size) and body size (measured as volume and centred and scaled) using Gaussian family linear mixed models following *daf‐2* RNAi exposure after resuming feeding following L1 arrest.
**Table S17:** Analysis of time at first egg production following L1 arrest recovery with a linear mixed effects model with Gaussian distribution: Time ~ Treatment + (1|Chamber) (Treatment: $\chi_{3}^{2}$ = 540.59, *p* < 0.0001). Treatments include: development only (daf_L1toL4), adulthood only (daf_L4), or lifelong (daf_L1).
**Table S18:** Analysis of population growth using a population index in semi‐natural conditions in populations founded by individuals following developmental *daf‐2* knockdown using a zero‐inflation negative binomial GLMM.
**Table S19:** Analysis of population growth using a population index in semi‐natural conditions in populations founded by individuals following developmental *daf‐2* knockdown using a zero‐inflation negative binomial GLMM for individual blocks.
**Table S20:** Analysis of population growth using a population index in semi‐natural conditions in populations founded by individuals following developmental + transient adulthood daf‐2 knockdown using a negative binomial GLMM.
**Table S21:** Analysis of population growth using a population index in semi‐natural conditions in populations founded by individuals following developmental + early‐adulthood daf‐2 knockdown using a negative binomial GLMM for individual blocks.
**Table S22:** Analysis of population growth using a population index in semi‐natural conditions in populations founded by individuals following transient adulthood daf‐2 knockdown using a zero‐inflation negative binomial GLMM.
**Table S23:** Analysis of population growth using a population index in semi‐natural conditions in populations founded by individuals following transient adulthood daf‐2 knockdown using a zero‐inflation negative binomial GLMM.
**Table S24:** Primer sequences are listed in the 5′ to 3′ direction. Primers were designed based on MIQE guidelines and taken from [1] for daf‐2 and [2] for actin‐3.
**Table S25:** Naturally co‐occurring bacterial strains from CeMBio added to compost in semi‐natural experiments.
**Figure S1:** Population index measured as the number of a individuals in a 5‐mL soil sample from a 250‐mL microcosm each containing a nematode population founded by 50 individuals following developmental *daf‐2* knockdown in individual blocks 1–4 (A–D, respectively). Populations were sampled weekly over a 21‐day period.
**Figure S2:** Population index measured as the number of a individuals in a 5‐mL soil sample from a 250‐mL microcosm each containing a nematode population founded by 50 individuals following developmental + early‐adulthood *daf‐2* knockdown in individual blocks 5 (A) and 6 (B). Populations were sampled weekly over a 21‐day period.
**Figure S3:** Population index measured as the number of a individuals in a 5‐mL soil sample from a 250‐mL microcosm each containing a nematode population founded by 50 individuals following transient adulthood *daf‐2* knockdown in individual blocks 1–5 (A–E, respectively). Populations were sampled weekly over a 21‐day period.
**Figure S4:** Average daily temperature during population growth assays in outdoor microcosms. Temperature conditions experienced by populations in outdoor microcosms following either (A) developmental *daf‐2* RNAi founder populations (four blocks across summer of 2023), (B) developmental + early adulthood *daf‐2* RNAi founder populations (two blocks in early summer of 2023), or (C) early adulthood *daf‐2* RNAi founder populations (blocks one through three in the summer and autumn of 2022 and blocks four and five in the early summer of 2023). Black lines show temperature fluctuations across experiments, with coloured boxes showing time periods for individual blocks.

## Data Availability

The data that support the findings of this study are openly available in Biological‐constraints‐during‐dev at https://github.com/irishsar92/Biological‐contraints‐during‐dev.
