# Supplementary material for: Live Fast, Die Old: Lowering Insulin Signalling During Development Can Speed Growth, Increase Reproduction and Lifespan, but Impair Environmental Sensing and Harm Offspring in Caenorhabditis elegans
Source: Aging Cell. 2026 Oct 23;25(11):e70783. doi: 10.1111/acel.70783 (PMC13694258; doi:10.1111/acel.70783)
Supplement: Supplementary file 1 [file ACEL-25-e70783-s001.docx]

**Supplementary Materials:**

| **Table S1:** Event history analysis with binomial GLMM for survival in nematodes treated with transient *daf-2* knockdown either during development, D1 of adulthood, or entire life with and without matricides censored | | | | | | | | | |
| --- | --- | --- | --- | --- | --- | --- | --- | --- | --- |
| **Matricides censored** | | | | | | | | | |
|  | *Est.* | | *SE* | | | *z* | | | *p* |
| (Intercept) | -1.081 | | 0.426 | | | -2.541 | | | 0.011 |
| Treatment.D1 | -2.048 | | 0.219 | | | -9.365 | | | <0.0001 |
| Treatment.Egg | -1.381 | | 0.193 | | | -7.153 | | | <0.0001 |
| Treatment.Lifelong | -3.349 | | 0.251 | | | -13.367 | | | <0.0001 |
| Random effects: | | | | Variance | | | Std. dev. | | |
| Plate_ID | | | | 0.0912 | | | 0.3018 | | |
| ID | | | | <0.0001 | | | 0.0001 | | |
| Day | | | | 7.5113 | | | 2.7407 | | |
| No. obs: 10498 Per group: Plate.ID: 38, ID: 410, Day: 53 | | | | | | | | | |
| **Type III Anova** | | *Χ^2^* | | | *df* | | | *p* | |
| (Intercept) | | 6.455 | | | 1 | | | 0.011 | |
| Treatment.ID | | 182.658 | | | 3 | | | <0.0001 | |
| **Matricides included** | | | | | | | | | |
|  | *Est.* | | *SE* | | | *z* | | | *p* |
| (Intercept) | -1.144 | | 0.385 | | | -2.971 | | | 0.003 |
| Treatment.D1 | -1.797 | | 0.209 | | | -8.616 | | | <0.0001 |
| Treatment.Egg | -1.154 | | 0.185 | | | -6.235 | | | <0.0001 |
| Treatment.Lifelong | -3.012 | | 0.239 | | | -12.626 | | | <0.0001 |
| Random effects: | | | | Variance | | | Std. dev. | | |
| Plate_ID | | | | 0.0876 | | | 0.2960 | | |
| ID | | | | <0.0001 | | | 0.0001 | | |
| Day | | | | 5.9686 | | | 2.4431 | | |
| No. obs: 10498 Per group: Plate.ID: 38, ID: 439, Day: 53 | | | | | | | | | |
| **Type III Anova** | | *Χ^2^* | | | *df* | | | *p* | |
| (Intercept) | | 8.826 | | | 1 | | | 0.003 | |
| Treatment.ID | | 163.327 | | | 3 | | | <0.0001 | |

**Table S2**: Cox proportional hazards analysis for survival in nematodes treated with daf-2 for the duration of development in 20°C with and without matricides censored.

| **Matricides censored** | | | | | |
| --- | --- | --- | --- | --- | --- |
|  | *Coefficient* | *Exp(coefficient)* | *SE* | | *p* |
| Treatment.daf-2 | -0.450 | 0.638 | 0.178 | | 0.012 |
| Random effect: | Plate.ID | Variance: 0.0001 | | SD: 0.0090 | |
| *Type II Anova* | Treatment: | *Χ^2^_1_ =* 6.382 | | p = 0.011 | |
|  |  |  | |  | |
| **Matricides included** | | | | | |
| Treatment.daf-2 | -0.346 | 0.708 | 0.169 | | 0.040 |
| Random effect: | Plate.ID | Variance: 0.033 | | SD: 0.182 | |
| *Type II Anova* | Treatment: | *Χ^2^_1_ =* 4.212 | | p = 0.040 | |

**Table S3**: Cox proportional hazards analysis for survival in nematodes treated with daf-2 for the duration of development in 15°C with and without matricides censored.

| **Matricides censored** | | | | | |
| --- | --- | --- | --- | --- | --- |
|  | *Coefficient* | *Exp(coefficient)* | *SE* | | *p* |
| Treatment.daf-2 | -2.354 | 0.095 | 0.336 | | <0.0001 |
| Random effect: | Plate.ID | Variance: 0.0004 | | SD: 0.1000 | |
| *Type II Anova* | Treatment: | *Χ^2^_1_ =* 49.083 | | p <0.0001 | |
|  |  |  | |  | |
| **Matricides included** | | | | | |
| Treatment.daf-2 | -0.979 | 0.376 | 0.211 | | <0.0001 |
| Random effect: | Plate.ID | Variance: 0.033 | | SD: 0.182 | |
| *Type II Anova* | Treatment: | *Χ^2^_1_ =* 21.493 | | p < 0.0001 | |

| **Table S4:** Cox proportional hazards analysis for survival in nematodes treated with *daf-2* for the duration of development in 25°C with and without matricides censored. | | | | | |
| --- | --- | --- | --- | --- | --- |
| **Matricides censored** | | | | | |
|  | *Coefficient* | *Exp(coefficient)* | *SE* | | *p* |
| *Matricides censored* | | | | | |
| Treatment.daf-2 | -0.056 | 0.945 | 0.356 | | 0.874 |
| Random effect: | Plate.ID | Variance: 0.4437 | | SD: 0.6661 | |
| *Type II Anova* | Treatment | *Χ^2^_1_ =* 0.025 | | p = 0.874 | |
|  |  |  | |  | |
| **Matricides included** | | | | | |
| Treatment.daf-2 | -0.049 | 0.952 | 0.255 | | 0.848 |
| Random effect: | Plate.ID | Variance: 0.1891 | | SD: 0.4349 | |
| *Type II Anova* | Treatment | *Χ^2^_1_ =* 0.037 | | p = 0.848 | |

**Table S5:** Anova results and estimated marginal means contrasts from linear model of the effect of RNAi treatment and time on normalised relative gene expression of *daf-2* in C. elegans up to 48 hours post removal from *daf-2* RNAi bacterial food.

| *Type III Anova* | | *Sum of sq.* | | *df* | | *F* | | *p* | |
| --- | --- | --- | --- | --- | --- | --- | --- | --- | --- |
| (Intercept) | | 0.0064 | | 1 | | 92.225 | | <0.0001 | |
| RNAi | | 0.0010 | | 1 | | 13.961 | | 0.0003 | |
| Hour | | 0.0076 | | 4 | | 27.404 | | <0.0001 | |
| RNAi*Hour | | 0.0004 | | 4 | | 1.403 | | 0.2347 | |
| Residuals | | 0.0126 | | 181 | |  | |  | |
| Residual SE = 0.008 on 181 df | | | | | | | | | |
|  | | | | | | | | | |
| *Contrasts for ev * daf-2* | | | | | | | | | |
| *Hour* | *Estimate* | | *SE* | | *df* | | *t* | | *p* |
| 0 | -0.010 | | 0.0027 | | 172 | | -3.740 | | 0.0002 |
| 12 | -0.013 | | 0.0027 | | 173 | | -4.723 | | <0.0001 |
| 24 | -0.006 | | 0.0027 | | 173 | | -2.366 | | 0.019 |
| 36 | -0.008 | | 0.0026 | | 172 | | -2.947 | | 0.004 |
| 48 | -0.004 | | 0.0028 | | 174 | | -1.566 | | 0.119 |

| **Table S6:** GLM for Gamma distribution with log link analysis of time to sexual maturity in nematodes after exposure to *daf-2* RNAi throughout development in 15, 20, and 25°C. | | | | | | | |
| --- | --- | --- | --- | --- | --- | --- | --- |
|  | *Est.* | | *SE* | | *z* | | *p* |
| **15°C** | | | | | | | |
| (Intercept) | 4.655 | | 0.003 | | 1502.57 | | <0.0001 |
| Treatment.daf | -0.025 | | 0.004 | | -5.561 | | <0.0001 |
| Residual deviance: 0.120 on 155 df | | | | | | | |
| Type II Anova | | Treatment: | | *Χ^2^_1_ =* 30.914 | | p < 0.0001 | |
| **20°C** |  | |  | |  | |  |
| (Intercept) | 4.084 | | 0.002 | | 1932.45 | | <0.0001 |
| Treatment.daf | -0.031 | | 0.003 | | -10.54 | | <0.0001 |
| Residual deviance: 0.422 on 442 df | | | | | | | |
| Type II Anova | | Treatment: | | *Χ^2^_1_ =* 111 | | p < 0.0001 | |
| **25°C** | | | | | | | |
| (Intercept) | 3.824 | | 0.002 | | 1725.99 | | <0.0001 |
| Treatment.daf | -0.008 | | 0.003 | | -2.461 | | 0.015 |
| Residual deviance: 0.121 on 224 df | | | | | | | |
| Type II Anova | | Treatment: | | *Χ^2^_1_ =* 6.055 | | p = 0.014 | |

**Table S7:** Model summary and Anova from a Gamma log link family model to analyse the effect of parental adulthood *daf-2* knockdown combined with offspring *daf-2* knockdown on developmental timing.

| **Experiment 1** | | | | | | | | |
| --- | --- | --- | --- | --- | --- | --- | --- | --- |
|  | Estimate | | | SE | | t | | p |
| (Intercept) | 4.094 | | | 0.004 | | 996.196 | | <0.0001 |
| Treatment.daf | -0.022 | | | 0.005 | | -4.744 | | <0.0001 |
| Parent.daf | 0.016 | | | 0.005 | | 3.309 | | 0.001 |
|  |  | | |  | |  | |  |
| **Type II Anova** | χ^2^_1_ | | | | | p | | |
| Treatment | 22.507 | | | | | <0.0001 | | |
| Parent Treatment | 10.950 | | | | | 0.001 | | |
|  |  | | |  | |  | |  |
| **Experiment 2** | | | | | | | | |
|  | Estimate | | | SE | | t | | p |
| (Intercept) | 3.703 | | | 0.006 | | 598.268 | | <0.0001 |
| Parent.daf | 0.059 | | | 0.009 | | 6.685 | | <0.0001 |
| Treatment.daf | 0.007 | | | 0.008 | | 0.929 | | 0.354 |
| Parent.daf*Treatment.daf | -0.025 | | | 0.011 | | -2.217 | | 0.027 |
|  |  | | |  | |  | |  |
| **Type III Anova** | χ^2^_1_ | | | | | p | | |
| Parent | 44.711 | | | | | <0.0001 | | |
| Treatment | 0.862 | | | | | 0.353 | | |
| Parent*Treatment | 4.915 | | | | | 0.027 | | |
|  |  | | | | |  | | |
|  | | | | | | | | |
| Contrasts (log scale) | Est. | | SE | | | t | p | |
| P0 ev + F1 ev: P0 daf-2 + F1 ev | | -0.059 | | | 0.009 | -6.685 | <0.0001 | |
| P0 ev + F1 ev: P0 ev + F1 daf-2 | | -0.007 | | | 0.008 | -0.929 | 0.790 | |
| P0 ev + F1 ev: P0 daf-2 + F1 daf-2 | | -0.041 | | | 0.008 | -5.081 | <0.0001 | |
| P0 daf-2 + F1 ev: P0 ev + F1 daf-2 | | 0.052 | | | 0.008 | 6.754 | <0.0001 | |
| P0 daf-2 + F1 ev: P0 daf-2 + F1 daf-2 | | 0.018 | | | 0.008 | 2.158 | 0.138 | |
| P0 ev + F1 daf-2: P0 daf-2 + F1 daf-2 | | -0.034 | | | 0.007 | -4.979 | <0.0001 | |

**Table S8:** Model summary and Anova output from analysis of age-specific reproduction for developmental daf-2 in 20°C using zero-inflation negative binomial GLMM. Model summary and Anova output from analysis of LRS and individual fitness from a general linear model.

| ***Age-specific reproduction*** | | | | | | | | | | | | | | |
| --- | --- | --- | --- | --- | --- | --- | --- | --- | --- | --- | --- | --- | --- | --- |
|  | | *Est.* | | | *SE* | | | | *t* | | | | *p* | |
| (Intercept) | | 2.971 | | | 0.111 | | | | 26.824 | | | | <0.0001 | |
| Treatment.ev | | -0.273 | | | 0.095 | | | | -2.862 | | | | 0.004 | |
| Day | | 1.389 | | | 0.078 | | | | 17.880 | | | | <0.0001 | |
| Day^2 | | -0.318 | | | 0.013 | | | | -25.103 | | | | <0.0001 | |
| Treatment.ev:Day | | 0.094 | | | 0.032 | | | | 2.971 | | | | 0.003 | |
| *Zero-inflation model ~ Day* | | | | | | | | | | | | | | |
| (Intercept) | | -9.411 | | | 1.034 | | | | -9.104 | | | | <0.0001 | |
| Day | | 1.752 | | | 0.221 | | | | 7.931 | | | | <0.0001 | |
| Random effects variance: | | | | | ID: <0.0001; SD <0.0001 | | | | | | | | | |
| *Type III Anova* | | | | | | | | | | | | | | |
|  | | | $x^{2}$ | | | | *df* | | | | *p* | | | |
| (Intercept) | | | 719.505 | | | | 1 | | | | <0.0001 | | | |
| Treatment | | | 8.192 | | | | 1 | | | | 0.004 | | | |
| Day | | | 319.699 | | | | 1 | | | | <0.0001 | | | |
| Day^2^ | | | 630.162 | | | | 1 | | | | <0.0001 | | | |
| Treatment*Day | | | 8.825 | | | | 1 | | | | 0.003 | | | |
| ***LRS*** | | | | | | | | | | | | | | |
|  | *Est.* | | | *SE* | | | | *t* | | | | *p* | | |
| (Intercept) | 262.864 | | | 5.153 | | | | 51.012 | | | | <0.0001 | | |
| Treatment.ev | -23.209 | | | 7.338 | | | | -3.163 | | | | 0.002 | | |
| *Type II Anova* | | | | | | | | | | | | | | |
|  | *Sum sq.* | | | | | *df* | | | | *F* | | | | *p* |
| Treatment | 29218 | | | | | 1 | | | | 10.003 | | | | 0.002 |
| Residuals | 627991 | | | | | 215 | | | |  | | | |  |
| ***Individual fitness (λ)*** | | | | | | | | | | | | | | |
|  | *Est.* | | | *SE* | | | | *t* | | | | *p* | | |
| (Intercept) | 4.044 | | | 0.046 | | | | 87.218 | | | | <0.0001 | | |
| Treatment.ev | 0.205 | | | 0.065 | | | | 3.145 | | | | 0.002 | | |
| *Type II Anova* |  |  |  |  |  |  |  |  |  |  |  |  |  |  |
|  | *Sum sq.* | | | | | *df* | | | | *F* | | | | *p* |
| Treatment | 2.339 | | | | | 1 | | | | 9.891 | | | | 0.002 |
| Residuals | 52.028 | | | | | 220 | | | |  | | | |  |

**Table S9:** Model summary and Anova output from analysis of age-specific reproduction following developmental daf-2 knockdown measured only in individuals that matured on or after the peak of maturation for controls using zero-inflation negative binomial GLMM. Model summary and Anova output from analysis of LRS and individual fitness from a general linear model.

| ***Age-specific reproduction*** | | | | | | | | | | | | | | |  |
| --- | --- | --- | --- | --- | --- | --- | --- | --- | --- | --- | --- | --- | --- | --- | --- |
|  | | *Est.* | | | *SE* | | | *z* | | | | *p* | | |  |
| (Intercept) | | 2.547 | | | 0.164 | | | 15.499 | | | | <0.0001 | | |  |
| Treatment.daf | | -0.009 | | | 0.076 | | | -0.119 | | | | 0.905 | | |  |
| Day | | 1.527 | | | 0.113 | | | 13.576 | | | | <0.0001 | | |  |
| Day^2 | | -0.317 | | | 0.018 | | | -17.173 | | | | <0.0001 | | |  |
| Zero-inflation model ~Day | | | | | | | | | | | | | | |  |
| (Intercept) | | -7.487 | | | 1.134 | | | -6.600 | | | | <0.0001 | | |  |
| Day | | 1.322 | | | 0.250 | | | 5.296 | | | | <0.0001 | | |  |
| Random effects variance: | | | | | ID <0.0001 | | | | | | | | | |  |
| *Type III Anova* | | | $x^{2}$ | | | | *df* | | | | *p* | | | |  |
| (Intercept) | | | 240.221 | | | | 1 | | | | <0.0001 | | | |  |
| Treatment | | | 0.014 | | | | 1 | | | | 0.905 | | | |  |
| Day | | | 184.305 | | | | 1 | | | | <0.0001 | | | |  |
| Day^2^ | | | 294.910 | | | | 1 | | | | <0.0001 | | | |  |
| ***LRS*** | | | | | | | | | | | | | | | |
|  | *Est.* | | | *SE* | | | | | *t* | | | | *p* | | |
| (Intercept) | 236.708 | | | 6.149 | | | | | 38.495 | | | | <0.0001 | | |
| Treatment.daf | 2.762 | | | 14.070 | | | | | 0.196 | | | | 0.845 | | |
| *Type II Anova* | *Sum sq.* | | | | | *df* | | | | *F* | | | | *p* | |
| Treatment | 105 | | | | | 1 | | | | 0.039 | | | | 0.845 | |
| Residuals | 236851 | | | | | 87 | | | |  | | | |  | |
| ***Individual fitness (λ)*** | | | | | | | | | | | | | | | |
|  | *Est.* | | | *SE* | | | | | *t* | | | | *p* | | |
| (Intercept) | 3.965 | | | 0.065 | | | | | 60.835 | | | | <0.0001 | | |
| Treatment.daf | -0.123 | | | 0.151 | | | | | -0.815 | | | | 0.418 | | |
| *Type II Anova* | *Sum sq.* | | | | | *df* | | | | *F* | | | | *p* | |
| Treatment | 0.209 | | | | | 1 | | | | 0.664 | | | | 0.418 | |
| Residuals | 27.972 | | | | | 89 | | | |  | | | |  | |

**Table S10:** Model summary and Anova output from analysis of age-specific reproduction for developmental daf-2 in 15°C using zero-inflation negative binomial GLMM. Model summary and Anova output from analysis of LRS and individual fitness from a general linear model.

| ***Age-specific reproduction*** | | | | | | | | | | | | | | | |
| --- | --- | --- | --- | --- | --- | --- | --- | --- | --- | --- | --- | --- | --- | --- | --- |
|  | | *Est.* | | | | | *SE* | | | *t* | | | | | *p* |
| (Intercept) | | 2.981 | | | | | 0.147 | | | 20.275 | | | | | <0.0001 |
| Treatment.daf | | 0.366 | | | | | 0.203 | | | 1.801 | | | | | 0.072 |
| Day^2^ | | -0.123 | | | | | 0.007 | | | -16.983 | | | | | <0.0001 |
| Day | | 0.719 | | | | | 0.069 | | | 10.442 | | | | | <0.0001 |
| Treatment.daf:Day^2^ | | 0.046 | | | | | 0.009 | | | 4.985 | | | | | <0.0001 |
| Treatment.daf:Day | | -0.278 | | | | | 0.092 | | | -3.027 | | | | | 0.002 |
| Zero-inflation model ~ Day | | | | | | | | | | | | | | | |
| (Intercept) | | -5.216 | | | | | 0.373 | | | -14.00 | | | | | <0.0001 |
| Day | | 0.547 | | | | | 0.053 | | | 10.38 | | | | | <0.0001 |
| Random effects variance: | | | | | | | ID: 0.076, SD: 0.276 | | | | | | | | |
| *Type III Anova* | | | | | | | | | | | | | | | |
|  | | | $X_{1}^{2}$ | | | | | | | |  | *p* | | | |
| (Intercept) | | | | | | 411.065 | | | | |  | <0.0001 | | | |
| Treatment | | | | | | 3.245 | | | | |  | 0.072 | | | |
| Day^2^ | | | | | | 288.439 | | | | |  | <0.0001 | | | |
| Day | | | | | | 109.027 | | | | |  | <0.0001 | | | |
| Treatment:Day^2^ | | | | | | 24.852 | | | | |  | <0.0001 | | | |
| Treatment:Day | | | | | | 9.166 | | | | |  | 0.002 | | | |
| ***LRS*** | | | | | | | | | | | | | | | |
|  | *Est.* | | | | *SE* | | | | *t* | | | | | *p* | |
| (Intercept) | 263.595 | | | | 6.237 | | | | 42.265 | | | | | <0.0001 | |
| Treatment.daf | 5.843 | | | | 9.158 | | | | 0.638 | | | | | 0.525 | |
| *Type II Anova* | | | | | | | | | | | | | | | |
|  | *Sum sq.* | | | *df* | | | | *F* | | | | | *p* | | |
| Treatment | 1172 | | | 1 | | | | 0.407 | | | | | 0.525 | | |
| Residuals | 391462 | | | 136 | | | |  | | | | |  | | |
| ***Individual fitness (λ)*** | | | | | | | | | | | | | | | |
|  | *Est.* | | | | *SE* | | | | *t* | | | | | *p* | |
| (Intercept) | 3.469 | | | | 0.042 | | | | 83.287 | | | | | <0.0001 | |
| Treatment.daf | 0.019 | | | | 0.060 | | | | 0.317 | | | | | 0.752 | |
| *Type II Anova* |  |  |  |  |  |  |  |  |  |  |  |  |  |  |  |
|  | *Sum sq.* | | | *df* | | | | *F* | | | | | *p* | | |
| Treatment | 0.011 | | | 1 | | | | 0.100 | | | | | 0.752 | | |
| Residuals | 13.324 | | | 120 | | | |  | | | | |  | | |

**Table S11:** Model summary and Anova output from analysis of age-specific reproduction for developmental daf-2 in 25°C using zero-inflation negative binomial GLMM. Model summary and Anova output from analysis of LRS and individual fitness from a general linear model.

| ***Age-specific reproduction*** | | | | | | | | | | | | | | |
| --- | --- | --- | --- | --- | --- | --- | --- | --- | --- | --- | --- | --- | --- | --- |
|  | | *Est.* | | | *SE* | | | | *z* | | | | *p* | |
| (Intercept) | | 4.181 | | | 0.179 | | | | 23.392 | | | | <0.0001 | |
| Treatment.daf | | -0.221 | | | 0.156 | | | | -1.413 | | | | 0.158 | |
| Day | | 0.729 | | | 0.146 | | | | 4.987 | | | | <0.0001 | |
| Day^2^ | | -0.365 | | | 0.029 | | | | -12.473 | | | | <0.0001 | |
| Treatment.daf:Day^2^ | | 0.160 | | | 0.068 | | | | 2.336 | | | | 0.020 | |
| *Zero-inflation model ~ Day* | | | | | | | | | | | | | | |
| (Intercept) | | -6.326 | | | 0.666 | | | | -9.104 | | | | <0.0001 | |
| Day | | 1.399 | | | 0.178 | | | | 7.931 | | | | <0.0001 | |
| Random effects variance: | | | | | ID: <0.0001; SD < 0.0001 | | | | | | | | | |
| *Type III Anova* | | | $x^{2}$ | | | | *df* | | | | *p* | | | |
| (Intercept) | | | 547.193 | | | | 1 | | | | <0.0001 | | | |
| Treatment | | | 1.996 | | | | 1 | | | | 0.158 | | | |
| Day | | | 24.870 | | | | 1 | | | | <0.0001 | | | |
| Day^2^ | | | 155.576 | | | | 1 | | | | <0.0001 | | | |
| Treatment*Day^2^ | | | 5.456 | | | | 1 | | | | 0.020 | | | |
| ***LRS*** | | | | | | | | | | | | | | |
|  | *Est.* | | | *SE* | | | | *t* | | | | *p* | | |
| (Intercept) | 182.278 | | | 6.908 | | | | 26.390 | | | | <0.0001 | | |
| Treatment.daf | 18.429 | | | 9.600 | | | | 1.920 | | | | 0.058 | | |
| *Type II Anova* | *Sum sq.* | | | | | *df* | | | | *F* | | | | *p* |
| Treatment | 9498 | | | | | 1 | | | | 3.685 | | | | 0.058 |
| Residuals | 283489 | | | | | 110 | | | |  | | | |  |
| ***Individual fitness (λ)*** | | | | | | | | | | | | | | |
|  | *Est.* | | | *SE* | | | | *t* | | | | *p* | | |
| (Intercept) | 4.496 | | | 0.079 | | | | 57.139 | | | | <0.0001 | | |
| Treatment.daf | 0.076 | | | 0.109 | | | | 0.698 | | | | 0.487 | | |
| *Type II Anova* | *Sum sq.* | | | | | *df* | | | | *F* | | | | *p* |
| Treatment | 0.163 | | | | | 1 | | | | 0.487 | | | | 0.487 |
| Residuals | 36.771 | | | | | 110 | | | |  | | | |  |

| **Table S12:** Model summary and Anova output from analysis of age-specific reproduction for adulthood only *daf-2* in 20°C using zero-inflation negative binomial GLMM. Model summary and Anova output from analysis of LRS and individual fitness from a general linear model. ***Age-specific reproduction*** | | | | | | | | | | | | | | |
| --- | --- | --- | --- | --- | --- | --- | --- | --- | --- | --- | --- | --- | --- | --- |
|  | | *Est.* | | | *SE* | | | | *z* | | | | *p* | |
| (Intercept) | | 10.248 | | | 0.119 | | | | 86.36 | | | | <0.0001 | |
| Treatment.ev | | -0.069 | | | 0.078 | | | | -0.88 | | | | 0.377 | |
| Day^2 | | -2.274 | | | 0.044 | | | | -52.20 | | | | <0.0001 | |
| Day | | -2.026 | | | 0.045 | | | | -45.51 | | | | <0.0001 | |
| Treatment.ev:Day | | 0.0311 | | | 0.035 | | | | 0.89 | | | | 0.371 | |
| *Zero-inflation model ~ Day* | | | | | | | | | | | | | | |
| (Intercept) | | -13.686 | | | 32.416 | | | | -0.422 | | | | 0.673 | |
| Day^2 | | 4.157 | | | 16.279 | | | | 0.255 | | | | 0.798 | |
| Random effects variance: | | | | | ID: <0.0001, OLRE: 0.0099 | | | | | | | | | |
| **Type III Anova** | | | $x^{2}$ | | | | *df* | | | | *p* | | | |
| (Intercept) | | | 7631.2771 | | | | 1 | | | | <0.0001 | | | |
| Treatment | | | 0.7814 | | | | 1 | | | | 0.377 | | | |
| Day | | | 27252.771 | | | | 1 | | | | <0.0001 | | | |
| Day^2^ | | | 2107.247 | | | | 1 | | | | <0.0001 | | | |
| Treatment*Day | | | 0.800 | | | | 1 | | | | 0.371 | | | |
| ***LRS*** | | | | | | | | | | | | | | |
|  | *Est.* | | | *SE* | | | | *t* | | | | *p* | | |
| (Intercept) | 267.979 | | | 4.161 | | | | 64.398 | | | | <0.0001 | | |
| Treatment.daf | -2.214 | | | 6.056 | | | | -0.366 | | | | 0.715 | | |
| *Type II Anova* | *Sum sq.* | | | | | *df* | | | | *F* | | | | *p* |
| Treatment | 220 | | | | | 1 | | | | 0.134 | | | | 0.715 |
| Residuals | 292821 | | | | | 178 | | | |  | | | |  |
| ***Individual fitness (λ)*** | | | | | | | | | | | | | | |
|  | *Est.* | | | *SE* | | | | *t* | | | | *p* | | |
| (Intercept) | 4.245 | | | 0.027 | | | | 157.110 | | | | <0.0001 | | |
| Treatment.daf | 0.025 | | | 0.040 | | | | 0.626 | | | | 0.532 | | |
| *Type II Anova* | *Sum sq.* | | | | | *df* | | | | *F* | | | | *p* |
| Treatment | 0.027 | | | | | 1 | | | | 0.391 | | | | 0.532 |
| Residuals | 11.939 | | | | | 174 | | | |  | | | |  |

| **Table S13:** Model summary and Anova output from analysis of age-specific reproduction following parental adulthood *daf-2* knockdown and offspring developmental *daf-2* knockdown using zero-inflation negative binomial GLMM. Model summary and Anova output from analysis of LRS and individual fitness from a general linear model. | | | | | | | | | | | | |
| --- | --- | --- | --- | --- | --- | --- | --- | --- | --- | --- | --- | --- |
|  | | *Est.* | | | *SE* | | | *z* | | | *p* | |
| (Intercept) | | 3.411 | | | 0.063 | | | 53.89 | | | <0.0001 | |
| Parent.daf | | 0.036 | | | 0.089 | | | 0.41 | | | 0.685 | |
| Treatment.daf | | 0.027 | | | 0.086 | | | 0.32 | | | 0.750 | |
| Day | | -34.271 | | | 2.043 | | | -16.78 | | | <0.0001 | |
| Day^2^ | | -20.029 | | | 1.756 | | | -11.41 | | | <0.0001 | |
| Parent.daf*Treatment.daf | | -0.481 | | | 0.128 | | | -3.76 | | | 0.0002 | |
| Parent.daf*Day | | -0.771 | | | 2.875 | | | -0.03 | | | 0.979 | |
| Parent.daf* Day^2^ | | -1.196 | | | 2.493 | | | -0.48 | | | 0.631 | |
| Treatment.daf*Day | | -3.129 | | | 2.774 | | | -1.13 | | | 0.259 | |
| Treatment.daf* Day^2^ | | 1.292 | | | 2.401 | | | 0.54 | | | 0.590 | |
| Parent.daf*Treatment.daf*Day | | -15.024 | | | 4.200 | | | -3.58 | | | 0.0003 | |
| Parent.daf*Treatment.daf*Day^2^ | | -10.483 | | | 3.574 | | | -2.93 | | | 0.003 | |
| *Zero-inflation model ~ Day* | | | | | | | | | | | | |
| (Intercept) | | -9.160 | | | 0.905 | | | -10.119 | | | <0.0001 | |
| Day | | 1.848 | | | 0.203 | | | 9.099 | | | <0.0001 | |
| Random effects variance: | | | | | ID: <0.0001, OLRE: 0.0099 | | | | | | | |
| **Type III Anova** | | | $x_{1}^{2}$ | | | | *df* | | *p* | | | |
| (Intercept) | | | 2903.844 | | | | 1 | | <0.0001 | | | |
| Parent | | | 0.164 | | | | 1 | | <0.0001 | | | |
| Treatment | | | 0.102 | | | | 1 | | <0.0001 | | | |
| Day^2^ | | | 354.385 | | | | 2 | | <0.0001 | | | |
| Parent*Treatment | | | 14.110 | | | | 1 | | 0.0002 | | | |
| Parent*Day^2^ | | | 0.236 | | | | 2 | | <0.0001 | | | |
| Treatment*Day^2^ | | | 1.867 | | | | 2 | | <0.0001 | | | |
| Parent*Treatment*Day^2^ | | | 16.527 | | | | 2 | | 0.0003 | | | |
| ***LRS*** | | | | | | | | | | | |  |
|  | *Est.* | | | *SE* | | *t* | | | | *p* | |  |
| (Intercept) | 281.633 | | | 5.544 | | 50.803 | | | | <0.0001 | |  |
| Parent.daf | 4.257 | | | 6.304 | | 0.675 | | | | 0.500 | |  |
| Offspring.daf | 17.896 | | | 6.309 | | 2.837 | | | | 0.005 | |  |
| *Type II Anova* | *Sum sq.* | | | *df* | | *F* | | | | *p* | |  |
| Parent | 797 | | | 1 | | 0.456 | | | | 0.500 | |  |
| Offspring | 14056 | | | 1 | | 8.046 | | | | 0.005 | |  |
| Residuals | 302217 | | | 173 | |  | | | |  | |  |
| ***Individual fitness (λ)*** | | | | | | | | | | | |  |
|  | *Est.* | | | *SE* | | *t* | | | | *p* | |  |
| (Intercept) | 4.467 | | | 0.043 | | 102.771 | | | | <0.0001 | |  |
| Parent.daf | 0.002 | | | 0.049 | | 0.032 | | | | 0.975 | |  |
| Offspring.daf | 0.189 | | | 0.049 | | 3.831 | | | | 0.0002 | |  |
| *Type II Anova* | *Sum sq.* | | | *df* | | *F* | | | | *p* | |  |
| Parent | 0.0001 | | | 1 | | 0.001 | | | | 0.975 | |  |
| Offspring | 1.576 | | | 1 | | 14.674 | | | | 0.0002 | |  |
| Residuals | 18.580 | | | 173 | |  | | | |  | |  |

**Table S14**: Proportion of dauers produced by parents with daf-2 knockdown from egg stage to early adulthood and EV control parents in high and medium food concentrations analysed using a binomial GLMM using glmer() with the cbind() function with number of individuals in dauer and number of individuals not in dauer as successes and failures, respectively.

|  | *Est.* | | *SE* | | *z* | | | *p* |
| --- | --- | --- | --- | --- | --- | --- | --- | --- |
| (Intercept) | 0.549 | | 0.202 | | 2.722 | | | 0.006 |
| Treatment.ev | -1.204 | | 0.238 | | -5.059 | | | <0.0001 |
| Food.low | -0.671 | | 0.237 | | -2.828 | | | 0.005 |
| *Type II Anova* | | | | | | | | |
|  | | $x^{2}$ | | *df* | | | *p* | |
| Treatment | | 25.597 | | 1 | | | <0.0001 | |
| Food | | 7.996 | | 1 | | | 0.005 | |
|  | |  | |  | | |  | |
| *Random effects:* | | *Variance* | | | | *Std. dev.* | | |
| Replicate:Population | | 0.090 | | | | 0.300 | | |
| Population | | 0.205 | | | | 0.453 | | |

| **Table S15**: Estimated relative population size in vials containing populations founded by parents that experienced *daf-2* knockdown from egg stage to early adulthood and control parents in high and medium food concentrations analysed using GLM with Gaussian error structure. | | | | |
| --- | --- | --- | --- | --- |
|  | Est. | SE | t | p |
| (Intercept) | 47.717 | 3.179 | 15.009 | <0.0001 |
| Treatment.ev | -3.633 | 3.671 | -0.990 | 0.336 |
| Food.low | -13.633 | 3.671 | -3.714 | 0.002 |
| *Type II Anova* | | | | |
|  | *Sum sq.* | *df* | *F* | *p* |
| Treatment | 66.01 | 1 | 0.980 | 0.336 |
| Food | 929.34 | 1 | 13.792 | 0.002 |
| Residuals | 1145.52 | 17 |  |  |

**Table S16:** Analysis of growth rates (measured at half of maximum size) and body size (measured as volume and centred and scaled) using Gaussian family linear mixed models following *daf-2* RNAi exposure after resuming feeding following L1 arrest.

| Growth rate (μm^3^/hr): Treatment Χ_3_^2^ = 0.648, p = 0.885 | | | | | |
| --- | --- | --- | --- | --- | --- |
|  | Est. | SE | df | t | p |
| (intercept) | 2.208 | 0.093 | 56.827 | 23.755 | <0.0001 |
| daf_L1 | 0.060 | 0.129 | 56.840 | 0.464 | 0.645 |
| daf_L1toL4 | -0.017 | 0.132 | 57.000 | -0.127 | 0.899 |
| daf_L4 | 0.070 | 0.132 | 57.289 | 0.530 | 0.598 |
| Random effects | | Variance | | SD | |
| Chamber | | 0.113 | | 0.337 | |
| Residual | | 0.228 | | 0.478 | |
|  |  |  |  |  |  |
| Maximum volume (μm^3^): Treatment Χ_3_^2^ = 8.324, p = 0.040 | | | | | |
|  | Est. | SE | df | t | p |
| (intercept) | -0.186 | 0.134 | 53.614 | -1.387 | 0.171 |
| daf_L1 | 0.537 | 0.187 | 53.722 | 2.878 | 0.006 |
| daf_L1toL4 | 0.310 | 0.190 | 53.902 | 1.632 | 0.108 |
| daf_L4 | 0.279 | 0.190 | 54.284 | 1.468 | 0.148 |
| Random effects | | Variance | | SD | |
| Chamber | | 0.216 | | 0.464 | |
| Residual | | 0.767 | | 0.876 | |

**Table S17:** Analysis of time at first egg production following L1 arrest recovery with a linear mixed effects model with Gaussian distribution: Time ~ Treatment + (1|Chamber) (Treatment: Χ_3_^2^ = 540.59, p < 0.0001). Treatments include: development only (daf_L1toL4), adulthood only (daf_L4), or lifelong (daf_L1)

|  | *Est.* | *SE* | *df* | *t* | *p* |
| --- | --- | --- | --- | --- | --- |
| (intercept) | 67.455 | 0.521 | 48.085 | 129.575 | <0.0001 |
| daf_L1 | 10.327 | 0.707 | 345.492 | 14.601 | <0.0001 |
| daf_L1toL4 | 13.405 | 0.766 | 346.986 | 17.510 | <0.0001 |
| daf_L4 | -0.504 | 0.692 | 347.434 | -0.728 | 0.467 |
| Random effects | | Variance | | SD | |
| Chamber | | 0.272 | | 0.521 | |
| Residual | | 23.686 | | 4.867 | |

| **Table S18:** Analysis of population growth using a population index in semi-natural conditions in populations founded by individuals following developmental *daf-2* knockdown using a zero-inflation negative binomial GLMM | | | | | | | |
| --- | --- | --- | --- | --- | --- | --- | --- |
|  | | *Estimate* | *SE* | | *z* | | *p* |
| Conditional Model ~ Treatment*Day*Block+ (1\|Population)+(1\|Plate_ID) | | | | | | | |
| (Intercept) | | 0.585 | 0.433 | | 1.350 | | 0.177 |
| Treatment.ev | | -1.813 | 0.602 | | -3.011 | | 0.003 |
| Day | | 0.134 | 0.022 | | 6.115 | | <0.0001 |
| Block2 | | 0.745 | 0.526 | | 1.415 | | 0.157 |
| Block3 | | 0.389 | 0.485 | | 0.803 | | 0.422 |
| Block4 | | 1.059 | 0.528 | | 2.006 | | 0.045 |
| Treatment.ev:Day | | 0.106 | 0.030 | | 3.550 | | 0.0004 |
| Treatment.ev:Block2 | | 0.567 | 0.740 | | 0.766 | | 0.443 |
| Treatment.ev:Block3 | | 2.280 | 0.674 | | 3.382 | | 0.0007 |
| Treatment.ev:Block4 | | 1.515 | 0.736 | | 2.058 | | 0.040 |
| Day:Block2 | | -0.045 | 0.026 | | -1.719 | | 0.086 |
| Day:Block3 | | -0.019 | 0.024 | | -0.773 | | 0.440 |
| Day:Block4 | | -0.074 | 0.027 | | -2.723 | | 0.006 |
| Treatment.ev:Day:Block2 | | -0.059 | 0.036 | | -1.614 | | 0.106 |
| Treatment.ev:Day:Block3 | | -0.139 | 0.034 | | -4.129 | | <0.0001 |
| Treatment.ev:Day:Block4 | | -0.110 | 0.037 | | -2.940 | | 0.003 |
|  | |  |  | |  | |  |
| Zero-inflation model ~ Day+Block | | | | | | | |
| (Intercept) | | 26.446 | 9874.52 | | 0.003 | | 0.998 |
| Day | | -3.628 | 1410.65 | | -0.003 | | 0.998 |
|  | |  |  | |  | |  |
| Dispersion model ~Block | | | | | | | |
| (Intercept) | | 1.241 | 0.157 | | 7.910 | | <0.0001 |
| Block2 | | 0.384 | 0.226 | | 1.702 | | 0.089 |
| Block3 | | -0.089 | 0.182 | | -0.489 | | 0.625 |
| Block4 | | -0.351 | 0.188 | | -1.872 | | 0.061 |
|  | |  |  | |  | |  |
| Random effects variance: | | Plate_ID: <0.0001, Population: 0.189 | | | | | |
| **Type III Anova** | $x^{2}$ | | | *df* | | *p* | |
| (Intercept) | 1.823 | | | 1 | | 0.177 | |
| Treatment | 9.064 | | | 1 | | 0.003 | |
| Day | 37.392 | | | 1 | | <0.0001 | |
| Block | 5.389 | | | 3 | | 0.145 | |
| Treatment*Day | 12.603 | | | 1 | | 0.0004 | |
| Treatment*Block | 17.521 | | | 3 | | 0.0006 | |
| Day*Block | 11.032 | | | 3 | | 0.012 | |
| Treatment*Day*Block | 21.818 | | | 3 | | <0.0001 | |

**Figure S1:** Population index measured as the number of a individuals in a 5-ml soil sample from a 250-ml microcosm each containing a nematode population founded by 50 individuals following developmental *daf-2* knockdown in individual blocks 1-4 (A – D, respectively). Populations were sampled weekly over a 21-day period.

| **Table S19**: Analysis of population growth using a population index in semi-natural conditions in populations founded by individuals following developmental *daf-2* knockdown using a zero-inflation negative binomial GLMM for individual blocks | | | | | | | | | | | | | | | | | | |  |  |  |
| --- | --- | --- | --- | --- | --- | --- | --- | --- | --- | --- | --- | --- | --- | --- | --- | --- | --- | --- | --- | --- | --- |
|  | | | $x^{2}$ | *Est.* | | *SE* | *z* | *p* | | | | *Est* | *SE* | | *z* | | *p* | | | |  |
| Block 1 | | |  |  | |  |  |  | | | | Zero-inflation model ~Day | | | | | | | | |  |
| (Intercept) | | 0.851 | | | -0.519 | 0.552 | -0.940 | | | 0.347 | | 2.271 | | 0.833 | | 2.728 | | 0.006 | | |  |
| Day | | 38.422 | | | 0.174 | 0.028 | 6.257 | | | <0.001 | | -0.311 | | 0.068 | | -4.564 | | <0.001 | | |  |
| Treatment | | 1.300 | | | -0.768 | 0.675 | -1.137 | | | 0.255 | |  | |  | |  | |  | | |  |
| Treat*Day | | 4.774 | | | 0.072 | 0.032 | 2.203 | | | 0.028 | |  | |  | |  | |  | | |  |
| Random effects variance: | | | | | | | | Plate_ID: 0.064; Population: 0.414 | | | | | | | | | | | |  |  |
| Block 2 | | |  |  | |  |  | | |  | | Zero-inflation model ~ Day | | | | | | | | |  |
| (Intercept) | | 15.687 | | | 1.313 | 0.332 | 3.961 | | | <0.001 | | 23.025 | | 3531.1 | | 0.007 | | 0.995 | | |  |
| Day | | 38.751 | | | 0.089 | 0.014 | 6.225 | | | <0.001 | | -3.109 | | 504.4 | | -0.006 | | 0.995 | | |  |
| Treatment | | 6.658 | | | -1.224 | 0.475 | -2.580 | | | 0.010 | |  | |  | |  | |  | | |  |
| Treat*Day | | 4.774 | | | 0.047 | 0.020 | 2.278 | | | 0.023 | |  | |  | |  | |  | | |  |
| Random effects variance: | | | | | | | | | Plate_ID: <0.0001; Population: 0.334 | | | | | | | | | | | | |
| Block 3 | | |  |  | |  |  | | |  | | Zero-inflation model ~ Day | | | | | | | | |  |
| (Intercept) | 19.626 | | | | 0.954 | 0.215 | 4.430 | | | <0.001 | | 14.917 | | 68.45 | | 0.218 | | 0.827 | | |  |
| Day | 107.551 | | | | 0.116 | 0.011 | 10.371 | | | <0.001 | | -2.040 | | 9.779 | | -0.209 | | 0.835 | | |  |
| Treatment | 2.505 | | | | 0.474 | 0.300 | 1.583 | | | 0.114 | |  | |  | |  | |  | | |  |
| Treat*Day | 4.534 | | | | -0.033 | 0.016 | -2.129 | | | 0.033 | |  | |  | |  | |  | | |  |
| Random effects variance: | | | | | | | | | | | Plate_ID: <0.0001; Population: 0.162 | | | | | | | | | | |
| Block 4 | | |  |  | |  |  | | |  | | Zero-inflation model ~ Day | | | | | | | | |  |
| (Intercept) | 56.515 | | | | 1.690 | 0.225 | 7.518 | | | <0.001 | | 11.667 | | 7.667 | | 1.522 | | 0.128 | | |  |
| Day | 26.116 | | | | 0.057 | 0.011 | 5.110 | | | <0.001 | | -1.479 | | 1.094 | | -1.352 | | 0.176 | | |  |
| Treatment | 4.109 | | | | -0.357 | 0.176 | 5.110 | | | 0.043 | |  | |  |  | |  | | | |  |
|  | | | Random effects variance: | | | | | Plate_ID: <0.0001; Population: 0.147 | | | | | | | | | | | |  |  |

**Table S20:** Analysis of population growth using a population index in semi-natural conditions in populations founded by individuals following developmental + transient adulthood daf-2 knockdown using a negative binomial GLMM

| *Model: ~ Treatment + Block*Day + (1\|Population) + (1\|Plate_ID)* | | | | | | | | |
| --- | --- | --- | --- | --- | --- | --- | --- | --- |
|  |  | Estimate | | SE | | Z | *p* | |
| (Intercept) |  | -0.905 | | 0.346 | | -2.616 | 0.009 | |
| Treatment.ev |  | 0.026 | | 0.344 | | 0.076 | 0.940 | |
| Day |  | 0.100 | | 0.014 | | 7.233 | <0.0001 | |
| Block6 |  | -3.923 | | 0.547 | | -7.173 | <0.0001 | |
| Day*Block6 |  | 0.192 | | 0.024 | | 7.863 | <0.0001 | |
| Random effects variance: | | | | Plate_ID: <0.0001, Population: 1.269 | | | | |
| **Type III Anova** | | | $x^{2}$ | | *df* | | | *p* |
| Treatment | | | 0.116 | | 1 | | | 0.734 |
| Day | | | 71.379 | | 1 | | | <0.0001 |
| Block | | | 15.801 | | 1 | | | <0.0001 |
| Day*Block | | | 14.460 | | 1 | | | 0.0001 |

**Table S21:** Analysis of population growth using a population index in semi-natural conditions in populations founded by individuals following developmental + early-adulthood daf-2 knockdown using a negative binomial GLMM for individual blocks

|  |  | $x^{2}$ | | | | *Estimate* | | *SE* | *Z* | *p* |
| --- | --- | --- | --- | --- | --- | --- | --- | --- | --- | --- |
| Block 5 | | | | | | | | | | |
| (Intercept) | | |  | 6.650 | | -0.766 | | 0.297 | -2.579 | 0.010 |
| Treatment (daf) | | |  | 1.635 | | -0.348 | | 0.272 | -1.279 | 0.201 |
| Day | | |  | 45.600 | | 0.106 | | 0.016 | 6.753 | <0.0001 |
| Random effects variance: | | | | | | | Population: 0.393, Plate_ID: <0.0001 | | | |
| Block 6 | | | | | | | | | | |
| (Intercept) | | |  | | 61.775 | -5.228 | | 0.668 | -7.821 | <0.0001 |
| Treatment (daf) | | |  | | 1.783 | 0.533 | | 0.829 | 0.644 | 0.520 |
| Day | | |  | | 73.757 | 0.016 | | 0.016 | 17.663 | <0.0001 |
| Random effects variance: | | | | | | | Population: 3.210, Plate_ID: <0.0001 | | | |

**Figure S2:** Population index measured as the number of a individuals in a 5-ml soil sample from a 250-ml microcosm each containing a nematode population founded by 50 individuals following developmental + early-adulthood *daf-2* knockdown in individual blocks 5 (A) and 6 (B). Populations were sampled weekly over a 21-day period.

**Table S22**: Analysis of population growth using a population index in semi-natural conditions in populations founded by individuals following transient adulthood daf-2 knockdown using a zero-inflation negative binomial GLMM

|  | | *Estimate* | *SE* | | *Z* | | *p* |
| --- | --- | --- | --- | --- | --- | --- | --- |
| Conditional Model ~ Treatment*Day+Block*Day+Treatment*Block+ (1\|Population)+(1\|Plate_ID) | | | | | | | |
| (Intercept) | | 0.020 | 0.324 | | 0.062 | | 0.95021 |
| Treatment.ev | | 0.591 | 0.309 | | 1.912 | | 0.05583 |
| Day | | 0.107 | 0.016 | | 6.678 | | <0.0001 |
| Block3 | | 1.057 | 0.369 | | 2.867 | | 0.004 |
| Block4 | | -2.699 | 0.412 | | -6.547 | | <0.0001 |
| Block5 | | 2.070 | 0.494 | | 4.192 | | <0.0001 |
| Block6 | | -4.921 | 0.823 | | -5.979 | | <0.0001 |
| Treatment.ev.:Day | | -0.018 | 0.011 | | -1.625 | | 0.104 |
| Day:Block3 | | 0.031 | 0.018 | | 1.767 | | 0.077 |
| Day:Block4 | | 0.144 | 0.019 | | 7.416 | | <0.0001 |
| Day:Block5 | | -0.156 | 0.025 | | -6.194 | | <0.0001 |
| Day:Block6 | | 0.186 | 0.040 | | 4.644 | | <0.0001 |
| Treatment.ev:Block3 | | -0.395 | 0.324 | | -1.219 | | 0.223 |
| Treatment.ev:Block4 | | -0.290 | 0.323 | | -0.897 | | 0.370 |
| Treatment.ev:Block5 | | -0.801 | 0.363 | | -2.204 | | 0.028 |
| Treatment.ev:Block6 | | 0.584 | 0.458 | | 1.275 | | 0.202 |
| Zero-inflation model ~ Day+Block | | | | | | | |
| (Intercept) | | 26.245 | 15595.52 | | 0.002 | | 0.999 |
| Block3 | | -4.786 | 1.513 | | -3.163 | | 0.002 |
| Block4 | | -2.695 | 1.195 | | -2.256 | | 0.024 |
| Block5 | | 0.793 | 0.421 | | 1.886 | | 0.059 |
| Block6 | | -1.183 | 1.374 | | -0.861 | | 0.389 |
| Day | | -3.622 | 2227.94 | | -0.002 | | 0.999 |
| Dispersion model ~Block | | | | | | | |
| (Intercept) | | 0.707 | 0.152 | | 4.652 | | <0.0001 |
| Block3 | | 0.029 | 0.180 | | 0.162 | | 0.871 |
| Block4 | | 0.613 | 0.246 | | 2.489 | | 0.013 |
| Block5 | | -0.170 | 0.242 | | -0.702 | | 0.483 |
| Block6 | | -0.749 | 0.357 | | -2.100 | | 0.036 |
|  | |  |  | |  | |  |
| Random effects: | | Plate_ID: | <0.0001 | | Population: | | 0.366 |
| *Type III Anova* | | | | | | | |
|  | $x^{2}$ | | | df | | p | |
| (Intercept) | -2.331 | | | 1 | | 1.000 | |
| Treatment | 0.035 | | | 1 | | 0.851 | |
| Day | -30.551 | | | 1 | | 1.000 | |
| Block | 24.477 | | | 4 | | <0.0001 | |
| Treatment*Day | 0.047 | | | 1 | | 0.828 | |
| Treatment*Block | 5.057 | | | 4 | | 0.282 | |
| Day*Block | 30.129 | | | 4 | | <0.0001 | |

**Figure S3:** Population index measured as the number of a individuals in a 5-ml soil sample from a 250-ml microcosm each containing a nematode population founded by 50 individuals following transient adulthood *daf-2* knockdown in individual blocks 1-5 (A – E, respectively). Populations were sampled weekly over a 21-day period.

**Table S23**: Analysis of population growth using a population index in semi-natural conditions in populations founded by individuals following transient adulthood daf-2 knockdown using a zero-inflation negative binomial GLMM

|  | | | |  | | Conditional model | | | | | |  | | | | | | |
| --- | --- | --- | --- | --- | --- | --- | --- | --- | --- | --- | --- | --- | --- | --- | --- | --- | --- | --- |
|  | | | $x^{2}$ | | | | *Est* | *SE* | | *z* | *p* | *Est.* | *SE* | | *z* | | *p* | |
| Block 1 | | | |  | |  | |  | |  |  | Zero-inflation model ~Day | | | | | | |
| (Intercept) | | 0.410 | | | | -0.198 | | 0.309 | | -0.641 | 0.522 | 22.083 | | 2705.65 | | 0.008 | | 0.993 |
| Day | | 75.478 | | | | 0.119 | | 0.014 | | 8.688 | <0.0001 | -3.042 | | 386.52 | | -0.008 | | 0.994 |
| Treatment | | 5.206 | | | | 0.986 | | 0.432 | | 2.282 | 0.023 |  | |  | |  | |  |
| Treat*Day | | 4.916 | | | | -0.043 | | 0.019 | | -2.217 | 0.027 |  | |  | |  | |  |
|  | Random effects variance: | | | | | | | | | Population/replicate: <0.0001; Population: 0.611 | | | | | | | | |
| Block 2 | |  | | |  | | |  | |  |  | Dispersion model ~ Day | | | | | | |
| (Intercept) | | 84.089 | | | | 2.305 | | 0.251 | | 9.170 | <0.0001 | -1.853 | | 0.281 | | -6.590 | | <0.001 |
| Day | | 33.490 | | | | 0.064 | | 0.011 | | 5.787 | <0.0001 | 0.194 | | 0.022 | | 8.755 | | <0.001 |
| Treatment | | 3.311 | | | | 0.568 | | 0.312 | | 1.820 | 0.069 |  | |  | |  | |  |
| Treat*Day | | 9.747 | | | | -0.039 | | 0.013 | | -3.122 | 0.002 |  | |  | |  | |  |
|  | Random effects variance: | | | | | | | | | Population/replicate: <0.0001; Population: 0.389 | | | | | | | | |
| Block 3 | |  | | |  | | |  | |  |  | Zero-inflation model ~ Day | | | | | | |
| (Intercept) | | 111.293 | | | | -2.207 | | 0.209 | | -10.55 | <0.0001 | 1.295 | | 0.582 | | 2.224 | | 0.026 |
| Day | | 677.786 | | | | 0.227 | | 0.009 | | 26.034 | <0.0001 | -0.284 | | 0.050 | | -5.685 | | <0.001 |
| Treatment | | 0.012 | | | | 0.020 | | 0.184 | | 0.011 | 0.914 |  | |  | |  | |  |
|  | Random effects variance: | | | | | | | | | Population/replicate: <0.0001; Population: 0.296 | | | | | | | | |
| Block 4 | |  | | |  | | |  | |  |  | Zero-inflation model ~ Day | | | | | | |
| (Intercept) | | 42.891 | | | 2.016 | | | 0.308 | | 8.297 | <0.0001 | 23.875 | | 2952.86 | | 0.008 | | 0.994 |
| Day | | 15.333 | | | -0.056 | | | 0.014 | | -3.916 | <0.0001 | -3.188 | | 421.837 | | -0.008 | | 0.994 |
| Treatment | | 1.312 | | | -0.323 | | | 0.282 | | -1.146 | 0.252 |  | |  | |  | |  |
|  | Random effects variance: | | | | | | | | | Population/replicate: <0.0001; Population: 0.233 | | | | | | | | |
| Block 5 | |  | | |  | | |  | |  |  |  | |  | |  |  | |
| (Intercept) | | 22.634 | | | -7.121 | | | 0.929 | | -7.666 | <0.0001 |  | |  | |  |  | |
| Day | | 12.967 | | | 0.384 | | | 0.035 | | 10.930 | <0.0001 |  | |  | |  |  | |
| Treatment | | 0.462 | | | 2.789 | | | 1.152 | | 2.422 | 0.015 |  | |  | |  |  | |
| Treat*Day | | 3.525 | | | -0.113 | | | 0.040 | | -2.798 | 0.005 |  | |  | |  |  | |
|  | Random effects variance: | | | | | | | | Population/replicate: <0.0001; Population: 3.155 | | | | | | | | | |

**Table S24**: Primer sequences are listed in the 5’ to 3’ direction. Primers were designed based on MIQE guidelines and taken from [1] for daf-2 and [2] for actin-3.

| Gene | Primer Sequences |
| --- | --- |
| *daf-2* | Forward: GTGGCGTGAGAATGAAGTGAG |
|  | Reverse: GGCTTATCGGCTACAATCGTC |
| *actin-3* | Forward: CCAAGAGAGGTATCCTTACCCTCAA |
|  | Reverse: AAGCTCATTGTAGAAGGTGTGATGC |

| **Table S25:** Naturally co-occurring bacterial strains from CeMBio added to compost in semi-natural experiments | | | | |
| --- | --- | --- | --- | --- |
| Strain | Strain Taxonomy | Source | Growth Time (agar) | Growth Time (LB broth) |
| JUb19 | Stenotrophomonas indicatrix | C. elegans N2 from mesocosm (Shapira) | 18 h in 37 C | Room temp on shaker for up to 36h |
| JUb66 | Lelliottia amnigena | rotting apple with wild C. elegans (Félix) | 18 h in 37 C | 18 h in 37C |
| MYb10 | Acinetobacter guillouiae | wild C. elegans from compost (Schulenburg) | Failed, grow in LB broth | Room temp on shaker for up to 36h |
| MYb11 | *Pseudomonas lurida* | wild C. elegans from compost (Schulenburg) | Failed, grow in LB broth | Room temp on shaker for up to 36h |
| MSPm1 | Pseudomonas berkeleyensis | C. elegans N2 from mesocosm (Shapira) | 18 h 37C | ~36 h in 37C |
| BIGb0172 | Comamonas piscis | wild C. elegans from rotting apple (Samuel) | Failed, grow in LB broth | Room temp on shaker for up to 36h |
| MYb71 | Ochrobactrum vermis | wild C. elegans from compost (Schulenburg) | 36-48 h in 37C | Room temp on shaker for up to 36h |
| JUb44 | Chryseobacterium scophthalmum | rotting apple with wild C. elegans (Félix) | 18 h in 37C | ~36 h in 37C |
| CEent1 | Enterobacter hormaechei | C. elegans N2 from mesocosm (Shapira) | 18 h in 37C | 18 h in 37C |
| OP50-1 | Escherichia coli | NA | 18 h in 37C | 18 h in 37C |

000

**B1**

**B3**

**B2**

**B4**

**B5**

**B2**

**B1**

**B1**

**B2**

**B3**

**B4**

**A**

**C**

**B**

**Figure S4 Average daily temperature during population growth assays in outdoor microcosms.** Temperature conditions experienced by populations in outdoor microcosms following either (**A**) developmental *daf-2* RNAi founder populations (four blocks across summer of 2023), (**B**) developmental + early adulthood *daf-2* RNAi founder populations (two blocks in early summer of 2023), or (**C**) early adulthood *daf-2* RNAi founder populations (blocks one through three in the summer and autumn of 2022 and blocks four and five in the early summer of 2023). Black lines show temperature fluctuations across experiments, with coloured boxes showing time periods for individual blocks

.

References:

1. Chauve, L. *et al.* High-throughput quantitative RT-PCR in single and bulk *C. elegans* samples using nanofluidic technology. *Journal of Visualized Experiments* **159**, e61132 (2020).
2. Akay, A. *et al.* The helicase Aquarius/EMB-4 is required to overcome intronic barriers to allow nuclear RNAi pathways to heritably silence transcription. *Dev Cell.* **42**, 241–255 (2017).
